# A Review on the Natural Products in Treatment of Diabetic Cardiomyopathy (DCM)

**DOI:** 10.31083/j.rcm2505165

**Published:** 2024-05-13

**Authors:** Pengyu Yao, Xiaoni Yang, Yun Qiao

**Affiliations:** ^1^Department of Traditional Chinese Medicine, Jinan Maternity and Child Care Hospital Affiliated to Shandong First Medical University, 250000 Jinan, Shandong, China; ^2^Department of Gerontology, The First Affiliated Hospital of Shandong First Medical University (Shandong Provincial Qianfoshan Hospital), 250014 Jinan, Shandong, China; ^3^Department of Traditional Chinese Medicine, Qilu Hospital of Shandong University, 250012 Jinan, Shandong, China

**Keywords:** diabetic cardiomyopathy (DCM), natural products, pharmacology, mechanism, efficacy, review

## Abstract

Diabetic cardiomyopathy is an insidious and fatal disease, imposing major financial and social burdens on affected individuals. Among the various methods proposed for the treatment of diabetic cardiomyopathy (DCM), treatments with natural products have achieved promising results due to their high efficiency and minimal side-effects. Literature was searched, analyzed, and collected using databases, including PubMed, Web of Science, Excerpt Medica, Science Direct, and Springer. In this study, we reviewed the DCM-related studies on 72 representative natural products. These natural products have been confirmed to be applicable in the therapeutic intervention of DCM, acting through various mechanisms such as the amelioration of metabolic abnormalities, protecting the mitochondrial structure and function, anti-oxidant stress, anti-inflammatory, anti-fibrosis, regulation of ${\mathrm{Ca}}^{2+}$ homeostasis and regulation of programmed cell death. The nuclear factor kappa B (NF-κB), nuclear factor erythroid 2-related factor 2 (Nrf-2), and transforming growth factor-β (TGF-β) have been extensively studied as high frequency signaling pathways for natural product intervention in DCM. The effectiveness of natural products in treating DCM has been revealed and studied, which provides a reference for DCM-specific drug discovery.

## 1. Introduction

Diabetic cardiomyopathy (DCM) is one of the most prevalent cardiovascular complications of diabetes mellitus (DM), which arises from the effects of type 1 diabetes mellitus (T1DM) and type 2 diabetes mellitus (T2DM) on the myocardium [1]. This disease process was first described by Rubler *et al*. in 1972 [2]. DCM is a specific cardiac manifestation in patients with diabetes, as a secondary effect of metabolic damage. It is characterized by gradual heart failure (HF) and detrimental cardiac remodeling (such as fibrosis, and diastolic and systolic dysfunction) [3]. The onset of DCM is insidious and often asymptomatic in the early stages of the disease. There is no efficient and specific methodology for DCM diagnosis at present, and one factor is the absence of symptoms [4]. Despite the presence of initial symptoms of DCM, such as mild left ventricular (LV) stiffness, slight decline in compliance, and diastolic dysfunction, patients frequently overlook them [5]. The myocardial interstitial fibrosis appears to be the initial detectable stage of DCM, and it is currently diagnosed mostly using cardiac magnetic resonance [6]. Once DCM is diagnosed, it is typically classified into two stages: in the early stages, left ventricular hypertrophy (LVH) and impaired diastolic function are present, while in the late stages, myocardial fibrosis, systolic dysfunction, and overt HF are present [7]. Changes in cardiac function in early stage DCM are reversible, especially when LVH has not yet occurred [6]. However, once systolic insufficiency occurs in patients with DCM, the prognosis becomes significantly worse. In late stage, changes of the metabolism with abnormal neurohumoral activation, and development of myocardial fibrosis could promote the coronary micro-circulation, then leading to diastolic function and systolic dysfunction in DCM [8].

The pathogenesis of DCM is likely to be complex and multi-factorial, has not yet been completely elucidated. Hyperglycemia (HG), insulin resistance, high free fatty acids (FFA), mitochondrial dysfunction, oxidative stress, myocardial inflammation, endothelial dysfunction, and calcium homeostasis are the basis of the pathogenesis of DCM, and these factors (independently or jointly) affect the occurrence and development of DCM. The heart is a primary target organ of the DM pathology, as HG is linked to an increased risk of diabetic cardiac events. Blood sugar control is the most fundamental measure in the treatment of DCM. The insulin deficiency and/or insulin resistance is the starting point of the series of reactions leading to impaired cardiac function in DCM, which is consistent with the pathogenesis of most diabetic complications. The healthy heart is metabolically flexible and can draw energy from diverse substrates. Fatty acid (FA) oxidation serves as the primary source of energy for the normal adult heart, accounting for approximately 60% [9]. HG and insulin deficiency and/or insulin resistance can lead to loss of metabolic flexibility in the heart. Cardiomyocytes are subject to a metabolic shift caused by HG and insulin deficiency and/or insulin resistance, which results in higher FA intake and β-oxidation to maintain enough levels of adenosine triphosphate (ATP) production [10]. However, over time, β-oxidation is incapable of properly processing all ingested FA, which causes loss of metabolic flexibility, intracellular lipid accumulation, and lipotoxicity. In recent years, the relationship between abnormal glycolipid metabolism and impaired cardiac function has become a hot topic in the study of DCM and other metabolic cardiomyopathies. The heart, which is the most metabolically active organ with the highest mitochondria content of any tissue, is extremely prone to oxidative distress [11]. Under normal physiological conditions of oxidative stress, oxidative phosphorylation of mitochondria in cardiomyocytes can produce 90% reactive oxygen species (ROS), and HG can promote ROS production in large quantities, which is one aspect of “glucotoxicity” in myocardial damage. In addition to being involved in oxidative stress, mitochondria also play other roles in DCM. In the diabetic heart, the mitochondria suffer from imbalanced dynamics, damaged biogenesis, and impaired mitophagy [12].

Myocardial inflammation is a heterogeneous process, partially contributes to structural and metabolic changes in the DM heart [13]. The chronic inflammatory response will appear in myocardial tissue, throughout the whole process of DCM. Endothelial dysfunction (ED) is involved in the pathological process of DCM, by promoting impaired myocardial metabolism, intracellular ${\mathrm{Ca}}^{2+}$ mishandling, endoplasmic reticulum (ER) stress, mitochondrial defect, accumulation of advanced glycation end-products (AGES), and extracellular matrix (ECM) deposition, leading to cardiac stiffness, fibrosis, and remodeling [14]. The precise regulation of calcium homeostasis in cardiomyocytes is a core link ensuring the systolic function of the heart. It has been strongly suggested in studies that several aspects related to ${\mathrm{Ca}}^{2+}$ handling are dysregulated in DCM, including altered expression and/or activity levels of the L-type ${\mathrm{Ca}}^{2+}$ channel activity, ryanodine receptor type 2 (RyR2), sarco/endoplasmic reticulum calcium ATPase (SERCA2a), and ${\mathrm{Na}}^{+}$/${\mathrm{Ca}}^{2+}$ exchanger (NCX) [15]. Multiple mechanisms contributing to the damage to the diabetic heart have been reported, but their complex relationships still need to be explored. In recent years, with the deepening and enrichment of research on DCM, this disease has been gradually recognized clinically, and has become a hot topic in the cross-research field relating to metabolic disease and heart disease.

DCM’s pathogenesis and clinical features have been well-studied in the past decade, but there are still few effective approaches for prevention and treatment [16]. At present, there is no effective drug for the treatment of DCM in clinical practice, so studying the research and development of effective therapeutic drugs is very necessary. Natural products continue to be a promising source of scaffolds with a wide range of structural diversity and bioactivity, that have the potential to be developed directly or used as starting points for optimizing novel drugs [17]. In recent years, natural products have been shown to be successful as anti-diabetic agents both *in vitro* and *in vivo*, as well as clinical trials [18, 19]. Considering the need for clinical treatment and scientific research focused on DCM, it is necessary to develop new drugs with high efficacy, few side-effects, and low prices. Natural products have been extensively discussed for their therapeutic effects, indicating their great potential for treating DCM. Over the past decade, a large number of studies have considered the use of natural products for the intervention of DCM, but the value of these research results still needs to be explored and sorted out. The purpose of this paper is to review the recent research progress concerning natural products and their underlying mechanisms of action, in order to provide a comprehensive introduction to the potential of natural products for the treatment of DCM.

## 2. Methods

We searched the databases PubMed, Web of Science, Excerpt Medica, Science Direct, and Springer for the period 2012 to 2022 regarding the use of natural products to treat DCM, using the following search terms: (“natural products” OR “effective constituents” OR “polysaccharides” OR “alkaloids” OR “flavonoids” OR “terpenoids” OR “phenylpropanoids” OR “quinones” OR “sterides” OR “glycosides”) AND (“diabetic cardiomyopathy” OR “DCM”).

**Fig. 1.**
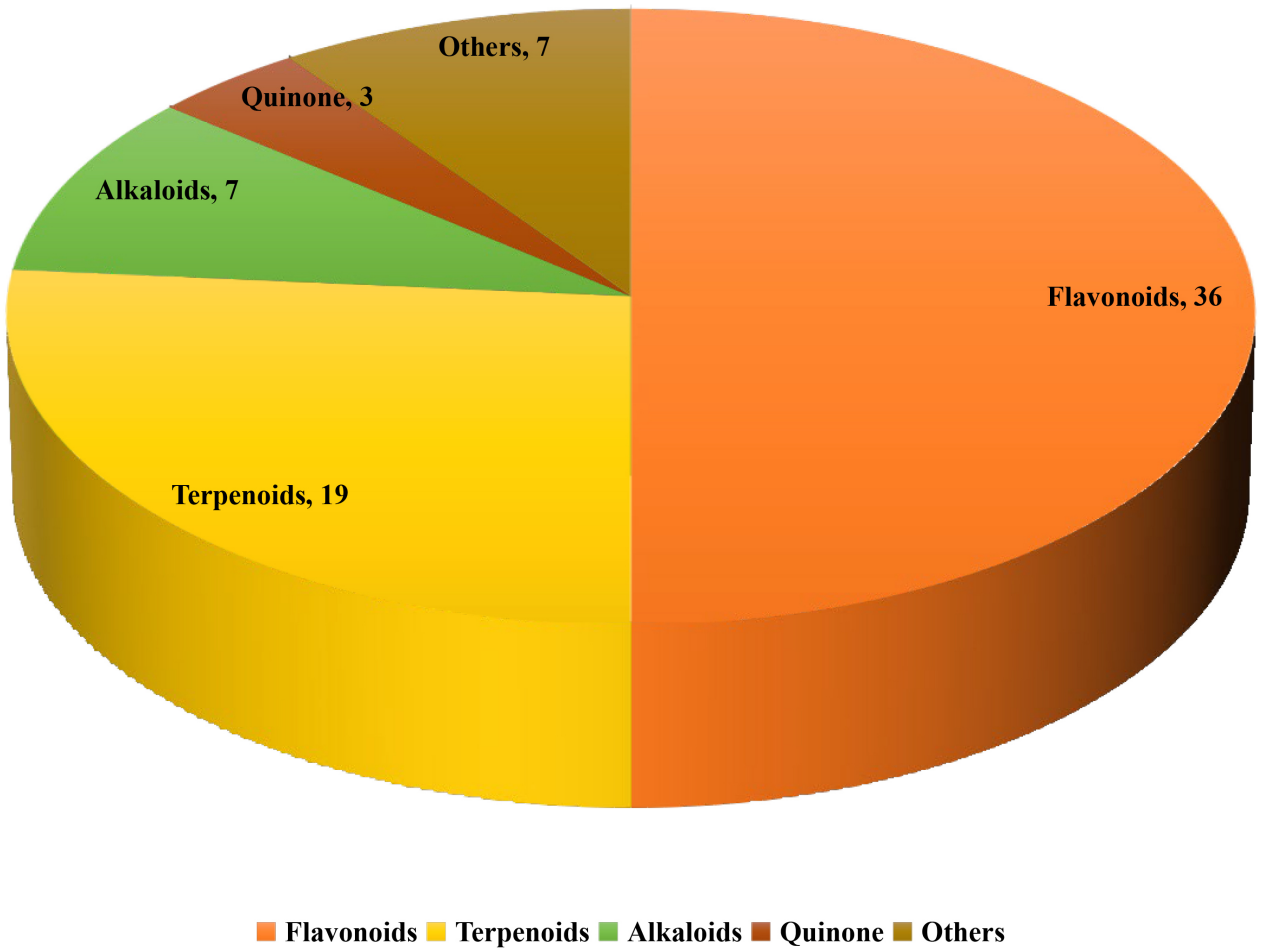
**Distribution of different sub-classes of natural products**.

This review excluded studies that were found to have significant methodological errors or lack scientific value. To help our classification efforts, studies that focused on mixtures of various compounds or crude extracts were also excluded from this study, in addition to those focused on natural products with poorly defined chemical structures; for example, Polysaccharides such as Astragalus polysaccharides and *Lycium barbarum* polysaccharides have also been shown to treat DCM *in vitro* and *in vivo*. However, since the chemical structure is unclear, we did not include them in the study. In total, 72 natural compounds were identified and grouped, based on their structural characteristics, into five categories: Flavonoids, terpenoids, alkaloids, quinones, and others. In the following, the different types of natural products are classified and introduced according to their relative quantities. Fig. 1 shows the numbers of the different types of natural products.

## 3. Flavonoids

**Fig. 2.**
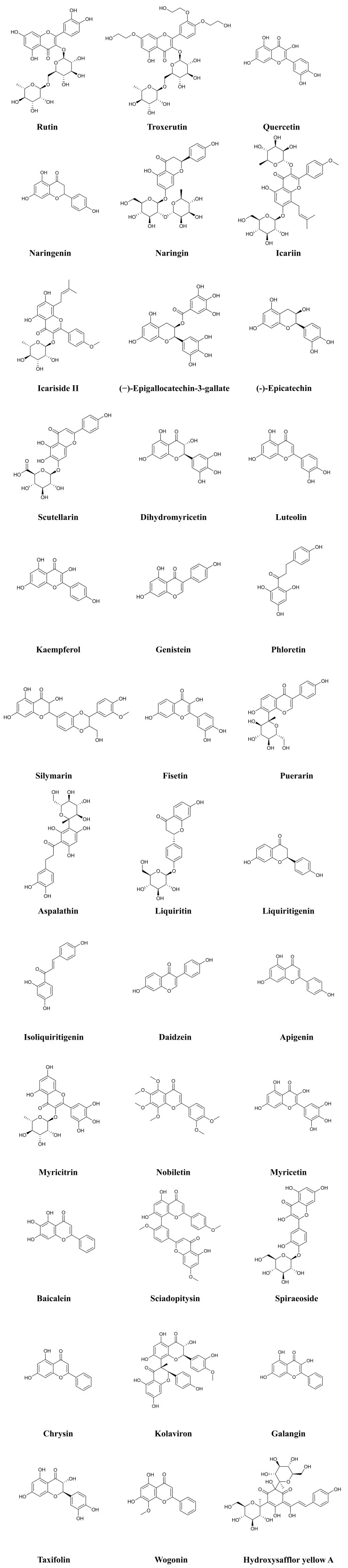
**Chemical structures of considered flavonoids**.

As one of the most diverse families of bioactive phytochemicals, flavonoids include over 9000 different compounds [20]. Flavonoid class compounds are naturally occurring poly-phenolic phytochemicals which are abundantly found among phytochemicals. Generally, the structure of flavonoids includes a basic C6–C3–C6 skeleton structure. There are many flavonoid sub-classes, such as flavonols, flavones, dihydroflavones, dihydroflavonols, chalcones, isoflavones, and biflavones. Flavonoids are often considered as breakthrough compounds for the development of new drugs, and have been widely studied for their effects in protecting the heart against diabetes-induced myocardial injury [21]. Flavonoids have the potential to alleviate DCM due to their anti-hyperglycemic, anti-oxidant, anti-inflammatory, and anti-apoptotic agents. A total of 36 flavonoids have been shown to possess effective therapeutic intervention effects on DCM, including 12 flavonols, 9 flavones, 4 dihydroflavones, 4 dihydroflavonols, 4 chalcones, 2 isoflavones, and 1 biflavone. Table 1 (Ref. [22, 23, 24, 25, 26, 27, 28, 29, 30, 31, 32, 33, 34, 35, 36, 37, 38, 39, 40, 41, 42, 43, 44, 45, 46, 47, 48, 49, 50, 51, 52, 53, 54, 55, 56, 57, 58, 59, 60, 61, 62, 63, 64, 65, 66, 67, 68, 69, 70, 71, 72, 73, 74, 75, 76, 77, 78, 79, 80, 81, 82, 83, 84, 85, 86, 87, 88, 89]) provides the basic information and mechanisms of 36 flavonoids from recent studies on DCM, while Fig. 2 shows the chemical structures of the 36 flavonoids.

**Table 1.** **Basic information and mechanisms of 36 flavonoids from recent studies on DCM**.

Number	Flavonoid Subclass	Compounds	Molecular formula	Weight (g/mol)	Resources	Animal/Cell model	Dosage (mg/kg/d; µm/24 h)	Dosing cycle	Target/Pathways/Mechanism	Effects	Reference
1	Flavonol	Rutin	$C_{27}$ $H_{30}$ $O_{16}$	610.5	*Ruta graveolens* L., *Scphora japonica* L., etc.	STZ-induced diabetic rats	8	12 days	/	Ameliorated metabolic abnormalities, anti-oxidative stress, anti-inflammatory, decreased myocardial apoptosis	[22]
						STZ-induced diabetic rats	50	24 weeks	/	Anti-inflammatory, anti-oxidative stress	[23]
						STZ-induced diabetic rats	50 (injected once a week)	7 weeks	/	Anti-oxidative stress, attenuates cardiac remodeling, improve left ventricular and myocardial dysfunction	[24]
						HFD + STZ-induced diabetic ApoE knockout mice	60	6 weeks	Up-regulated Akt and MAPK signaling pathway	Decreased myocardial fibrosis, increased myocardial capillary density and decreased apoptosis, reduced ectopic lipid deposition, anti-oxidative stress	[25]
						Alloxan-induced diabetic rats	100	4 weeks	/	Decreased myocardial fibrosis, inhibition of metabolic acidosis	[26]
						STZ-induced diabetic rats	5 and 40	4 weeks	/	Suppression of tTG	[27]
						HG-induced H9c2 myoblast cells	2, 10, and 50	24 h	/	Decreased myocardial apoptosis, inhibits endoplasmic reticulum stress	[28]
2	Flavonol	Troxerutin	$C_{33}$ $H_{42}$ $O_{19}$	742.7	*Robinia pseudoacacia* L.	STZ-induced diabetic rats	150	4 weeks	Activation of the AKT/IRS/JNK signaling pathway	Regulate glucose and lipid metabolism disorders, reduces levels of ROS	[29]
3	Flavonol	Quercetin	$C_{15}$ $H_{10}$ $O_{7}$	302.23	*Psidii guajavae* L.*, Allium cepa* L.*, Camellia sinensis* (L.) Kuntze, *Malus pumila* Mill., etc.	HC-induced diabetic rats	0.005	4 weeks	/	Anti-oxidant stress, protected against diastolic dysfunction, prevent cholesterol accumulation and ATP reduction	[30]
						HFD + STZ-induced diabetic mice	100	4 months	/	Anti-inflammatory, regulated glycerophospholipid metabolism, ameliorate cardiac dysfunction, decreased myocardial fibrosis	[31]
						Zucker Diabetic Fatty rats (fa/fa)	20	6 weeks	Attenuated pro-hypertrophic gene transcription-regulated HDAC4/MEF2 signaling pathway, mitigation of pro-hypertrophic NFAT/calcineurin network	Improved diastolic dysfunction, reduced LV collagen content, reduced LV mass thickness and increased the internal diameter of LV	[32]
						High-fat feeding + STZ-induced diabetic rats; HG-induced H9c2 cardiomyocytes	160	6 months	Activation of Nrf-2 signaling pathway	Inhibited myocardial fibrosis, reduced the accumulation of ROS, inhibited pyroptosis	[33]
						STZ-induced diabetic rats	10 and 30	28 days	/	Anti-oxidative stress, decreased cardiomyocyte apoptosis	[34]
						TAC-induced congestive heart failure mice	50	15 days	Promote the desuccinylation of IDH2 through SIRT5	Maintain mitochondrial homeostasis, anti-inflammatory, decreased myocardial fibrosis	[35]
4	Dihydroflavone	Naringenin	$C_{15}$ $H_{12}$ $O_{5}$	272.25	Citrus × aurantium, *Citrus maxima* (Burm.) Merr., *Citrus reticulata* Blanco	STZ-induced diabetic mice	25 and 75	4 weeks	Activation of PPARs.	Anti-cardiac hypertrophy	[36]
						HG-H9c2 cells	0.1, 1, and 10	48 h	Activation of EETs/PPARs	Anti-cardiac hypertrophy	[37]
						STZ-induced diabetic mice; HG- H9c2 cells	25, 50, and 75; 10	63 days; 2 h	Inhibition of NF-κB signaling pathway, activation of Nrf-2 signaling pathway	Anti-oxidative stress, anti-inflammatory, decreased myocardial fibrosis and alleviated cardiomyocyte apoptosis	[38]
5	Dihydroflavone	Naringin	$C_{27}$ $H_{32}$ $O_{14}$	580.5	Citrus × aurantium, *Citrus maxima* (Burm.) Merr., *Citrus reticulata* Blanco	HS + HFD + STZ-induced diabetic rats; H9c2 cardiac cells	25, 50 and 100; 80	6 weeks; 2 h	Up-regulated KATP channels, inhibition of the NF-κB signaling pathway	Decreased cardiomyocyte apoptosis, anti-oxidative stress	[39]
						Diabetic db/db mice	20, 40 and 60	4 weeks	/	Anti-inflammatory, anti-oxidative stress, decreased myocardial fibrosis	[40]
						STZ-induced diabetic rats	25, 50 and 100	8 weeks	/	Anti-oxidative stress, decreased cardiac apoptosis	[41]
6	Flavonol	Icariin	$C_{33}$ $H_{40}$ $O_{15}$	676.7	*Epimedium brevicornu* Maxim.	Diabetic db/db mice; primary cardiomyocyte obtained from the ventricles of newborn C57 mice	30	16 weeks	Activation of Apelin/SIRT3 signaling pathway	Rescued the impaired mitochondria, decreased cardiac apoptosis	[42]
						STZ + HG/high-fat diet-induced diabetic rats	60 and 120	12 weeks	Suppression of TGF-β1/Smad signaling pathway	Improved glucose tolerance and insulin sensitivity, inhibited ECM accumulation, decreased myocardial fibrosis	[43]
						STZ + HG/high-fat diet-induced diabetic rats	30 and 60	8 weeks	Suppression of NOS3/PDE5-sGC-cGMP-PKG signaling pathway	Improves myocardial functions, decreased myocardial fibrosis, improve ${\mathrm{Ca}}^{2+}$ hyperactivities and dysfunction	[44]
7	Flavonol	Icariside II	$C_{27}$ $H_{30}$ $O_{10}$	514.5	*Epimedium pubescens*, *Epimedium grandiflorum*	STZ-induced diabetic rats	5	8 weeks	Activation the Akt/NOS/NF-κB signaling pathway	Anti-inflammatory, anti-oxidative stress, decreases cardiac apoptosis	[45]
8	Flavonol	(−)-Epigallo-catechin-3-gallate	$C_{22}$ $H_{18}$ $O_{11}$	458.4	Tea	STZ-induced diabetic rats/HG-induced H9c2 cardiac cells	100; 20	6 weeks; 24 h	Stimulating the SIRT1 signaling pathway	Attenuated cardiac dysfunction, decreased myocardial fibrosis, decreased myocardial apoptosis, anti-oxidative stress	[46]
						HFD + STZ-induced diabetic rats	40 and 80	8 weeks	Regulation of AMPK/mTOR signaling pathway, repression of the TGF-β/MMPs signaling pathway	Activation of autophagy, decreased myocardial fibrosis	[47]
						STZ-nicotinamide-induced diabetic rats	2 (on alternate days)	1 month	/	Anti-inflammatory, anti-oxidative stress, decreased myocardial fibrosis, decreased cardiac apoptosis	[48]
9	Dihydroflavonol	(-)-Epicatechin	$C_{15}$ $H_{14}$ $O_{6}$	290.27	Tea	Hyperglycemia-induced cardiac fibroblasts	1	48 h	Regulation of Smad/TGF-β1 signaling pathway	Decreased myocardial fibrosis	[49]
10	Flavone	Scutellarin	$C_{21}$ $H_{18}$ $O_{12}$	462.4	*Scutellaria baicalensis* Georgi	STZ-induced diabetic mice	10 and 20	4 weeks	/	Anti-inflammatory, anti-oxidative stress, decreased myocardial fibrosis	[50]
						HFD + STZ-induced diabetic rats	10 and 20	6 weeks	Regulation of Nrf-2/Keap1 signaling pathway and TLR4/MyD88/NF-κB signaling pathway	Anti-inflammatory, anti-oxidative stress, decreased myocardial fibrosis, decreased cardiac apoptosis	[51]
						HS + HFD + STZ-induced diabetic rats	100 and 200	8 weeks	/	Activation of autophagy, decreased cardiac apoptosis	[52]
11	Dihydroflavonol	Dihydromyricetin	$C_{15}$ $H_{12}$ $O_{8}$	320.25	*Ampelopsis grossedentata* Hand.-Mazz.	STZ-induced diabetic rats	100	2 weeks	Suppression of miR-34a	Activation of autophagy, mitigates cardiac dysfunction	[53]
						STZ-induced diabetic mice	100	14 weeks	Activation of AMPK/ULK1 signaling pathway	Anti-inflammatory, anti-oxidative stress, decreased cardiac apoptosis, improved mitochondrial function, restored cardiac autophagy	[54]
						STZ-induced diabetic mice	250	12 weeks	Activation of SIRT3	Improved cardiac dysfunction; ameliorated cardiac hypertrophy, anti-inflammatory, anti-oxidative stress, decreased myocardial fibrosis, decreased cardiac necroptosis	[55]
12	Flavone	Luteolin	$C_{15}$ $H_{10}$ $O_{6}$	286.24	*Capsicum annuum* L., *Lonicera japonica* Thunb., *Perilla frutescens* (L.) Britton, etc.	STZ-induced diabetic mice; HG-induced H9C3 cells	20; 5 and 10	15 weeks; 24 h	Inhibition of NF-κB signaling pathway, activation Nrf-2 signaling pathway	Anti-oxidant stress, anti-inflammatory, decreased myocardial fibrosis and hypertrophy	[56]
						STZ-induced diabetic mice and db/db mice; HG and high-insulin-induced primary neonatal rat cardiomyocytes	20; 1 µM	12 weeks; 48 h	Up-regulated phosphorylated protein AMPK and AKT/GSK-3 signaling pathway	Prevented cardiac hypertrophy, decreased myocardial fibrosis, ameliorate cardiac dysfunction	[57]
						STZ-induced diabetic rats	200	8 weeks	/	Anti-oxidative stress, inhibits left ventricular dysfunction and remodeling	[58]
13	Flavonol	Kaempferol	$C_{15}$ $H_{10}$ $O_{6}$	286.24	Tea, Citrus × aurantium, *Malus pumila* Mill., etc.	STZ-induced diabetic mice; HG-induced H9c2 cells	10; 2.5	8 weeks; 1 h	Inhibition of NF-κB signaling pathway, activation of Nrf-2 signaling pathway	Anti-inflammatory, anti-oxidative stress, decreased myocardial fibrosis, decreased cardiac apoptosis	[59]
						STZ-induced diabetic rats	50	8 weeks	Activation of SIRT1 signaling pathway, down-regulated TGF-β1, up-regulated Nrf-2, and suppression of NF-κB p65	Anti-inflammatory, anti-oxidative stress, decreased myocardial fibrosis, inhibits cardiomyocytes intrinsic cell death	[60]
14	Flavone	Genistein	$C_{15}$ $H_{10}$ $O_{5}$	270.24	*Glycine max* (L.) Merr., *Pueraria montana var. lobata* (Ohwi) Maesen & S. M. Almeida	STZ-induced diabetic rats	300	24 weeks	/	Decreased myocardial fibrosis and hypertrophy	[61]
						STZ-induced diabetic rats	5 and 25	4 weeks	Suppression of the TGF-β1/Smad3 signaling pathway	Decreased myocardial fibrosis and hypertrophy	[62]
15	Chalcone	Phloretin	$C_{15}$ $H_{14}$ $O_{5}$	274.27	*Malus pumila* Mill., *Litchi chinensis* Sonn.	STZ-induced diabetic mice; HG-induced H9c2 cells	20	56 days; 24 h	Restored SIRT1 expression	Decreased myocardial fibrosis, anti-inflammatory	[63]
						STZ-induced diabetic mice; HG-induced H9c2 cells	10 (on two days); 10	8 weeks; 1 h	Activation of Keap1/Nrf-2 signaling pathway	Anti-oxidative stress, decreased myocardial fibrosis	[64]
16	Dihydroflavonol	Silymarin	$C_{25}$ $H_{22}$ $O_{10}$	482.4	*Silybum marianum* (L.) Gaertn.	STZ-induced diabetic rats; primary cardiac fibroblasts	25, 50, and 100; 100 mmol/L	5 weeks; 24 h	Inhibition of TGF-β1/Smad signaling pathway	Decreased myocardial fibrosis and collagen deposition	[65]
						Alloxan-induced diabetic rats	120	10 days	/	Decreased cardiac apoptosis	[66]
17	Flavonol	Fisetin	$C_{15}$ $H_{10}$ $O_{6}$	286.24	*Acacia greggii* A.Gray, *Vachellia farnesiana* (L.) Wight & Arn., etc.	STZ-induced diabetic rats	2.5	6 weeks	/	Anti-inflammatory, anti-oxidative stress, decreased cardiac apoptosis, ameliorates hyperglycemia and dyslipidemia	[67]
						STZ-induced diabetic rats	2.5	12 weeks	/	Anti-inflammatory, anti-oxidative stress	[68]
18	Isoflavone	Puerarin	$C_{21}$ $H_{20}$ $O_{9}$	416.4	*Pueraria montana var. lobata* (Ohwi) Maesen & S. M. Almeida	STZ-induced diabetic rats; HG-induced H9c2 cells	50, 100, and 200; (${\mathrm{10}}^{-\,4}$, ${\mathrm{10}}^{-\,5}$, ${\mathrm{10}}^{-\,6}$ mol/L)	8 weeks; 12 h	Inhibition of NF-κB signaling pathway	Anti-inflammatory, decreased myocardial fibrosis	[69]
						STZ-induced diabetic rats	50, 100, 200	6 weeks	/	Regulated lipid metabolism disorder, anti-inflammatory, anti-oxidative stress, decreased myocardial fibrosis, preserved the myocardial integrity, inhibited pyroptosis	[70]
19	Chalcone	Aspalathin	$C_{21}$ $H_{24}$ $O_{11}$	452.4	*Aspalathus linearis*	Diabetic db/db mice; HG-induced H9c2 cells	13 and 130; 1µM	6 weeks; 6 h	Activation of Nrf-2	Anti-inflammatory, anti-oxidative stress, decreased cardiac apoptosis	[71]
						HG-induced H9c2 cells	6 µM	6 h	/	Anti-inflammatory, anti-oxidative stress, decreased cardiac apoptosis	[72]
20	Dihydroflavone	Liquiritin	$C_{21}$ $H_{22}$ $O_{9}$	418.4	*Glycyrrhiza uralensis* Fisch.	High fructose-induced diabetic mice; cardiomyocytes from the experimental mice	10 and 20; 0–32 µM	10 weeks; 24 h	Suppression of NF-κB and MAPKs signaling pathways	Anti-inflammatory, decreased myocardial fibrosis	[73]
21	Dihydroflavone	Liquiritigenin	$C_{15}$ $H_{12}$ $O_{4}$	256.25	*Glycyrrhiza uralensis* Fisch.	High fructose-induced mice as the experimental mice; fructose-induced H9c2 cells	4, 8 and 16; 10 and 20	10 weeks; 24 h	Inhibition of NF-κB signaling pathway	Anti-inflammatory, decreased myocardial fibrosis	[74]
22	Chalcone	Isoliquiritigenin	$C_{15}$ $H_{12}$ $O_{4}$	256.25	*Glycyrrhiza uralensis* Fisch.	STZ-induced diabetic mice; HG-induced H9c2 cells	10 and 20; 2.5, 5, 10, 20, and 40	12 weeks; 24 h	Inhibition of MAPKs signaling pathway, induction of the Nrf-2 signaling pathway	Suppression HG-induced hypertrophy, anti-inflammatory, decreased myocardial fibrosis, decreased cardiac apoptosis	[75]
23	Isoflavone	Daidzein	$C_{15}$ $H_{10}$ $O_{4}$	254.24	*Glycine max* (L.) Merr.	STZ-induced diabetic rats	25, 50, and 100	4 weeks	/	Anti-oxidative stress	[76]
24	Flavone	Apigenin	$C_{15}$ $H_{10}$ $O_{5}$	270.24	*Apium graveolens* L., *Verbena officinalis* L., etc.	STZ-induced diabetic mice; HG-induced H9c2 cells	100; 25	7 months; 24 h	Inhibition of NF-κB/P65 signaling pathway	Anti-inflammatory, anti-oxidative stress, decreased myocardial fibrosis, decreased cardiac apoptosis	[77]
25	Flavonol	Myricitrin	$C_{21}$ $H_{20}$ $O_{12}$	464.4	*Morella rubra* Lour.	STZ-induced diabetic mice; AGEs-induced H9c2 cells	75, 150, and 300; 3.12, 6.25, 12.5, 25, 50, and 100 µg/mL	8 weeks; 12 h	Activation of the PI3K/Akt signaling pathway and Nrf-2/ARE signaling pathway	Anti-inflammatory, anti-oxidative stress, decreased myocardial fibrosis, decreased cardiac apoptosis, attenuates hypertrophic	[78]
26	Flavone	Nobiletin	$C_{21}$ $H_{22}$ $O_{8}$	402.4	Citrus × aurantium, *Citrus maxima* (Burm.) Merr., *Citrus reticulata* Blanco	STZ-induced diabetic mice	50	11 weeks	Suppression of JNK, P38, and NF-κB signaling pathways	ameliorated oxidative stress, inflammatory status and apoptosis, decreased myocardial fibrosis	[79]
27	Flavonol	Myricetin	$C_{15}$ $H_{10}$ $O_{8}$	318.23	*Morella rubra* Lour.	STZ-induced diabetic mice	200	6 months	Inhibition of IκBα/NF-κB and enhancing Nrf-2/HO-1 signaling pathways	Anti-inflammatory, anti-oxidative stress, decreased myocardial fibrosis, decreased cardiac apoptosis	[80]
28	Flavone	Baicalein	$C_{15}$ $H_{10}$ $O_{5}$	270.24	*Scutellaria baicalensis* Georgi	HS + HFD + STZ-induced diabetic rats	100 and 200	16 weeks	Activation of PI3K/Akt signaling pathway	Anti-inflammatory, anti-oxidative stress	[81]
29	Flavone	Sciadopitysin	$C_{33}$ $H_{24}$ $O_{10}$	580.5	*Ginkgo biloba* L.	HG-induced Human cardiomyocyte line AC16	0, 1, 5, 10, and 20 µM	24 h	Activation of PI3K/PKB/GSK‐3β signaling pathway	Anti-oxidative stress, decreased cardiac apoptosis	[82]
30	Flavonol	Spiraeoside	$C_{21}$ $H_{20}$ $O_{12}$	464.4	*Spiraea salicifolia* L.	HG‐induced human cardiomyocytes; HG-induced AC16 cells	1, 5, 10, and 20 µM	0, 24, and 48 h	Activation of PI3K/Akt/Nrf-2 pathway	Anti-oxidative stress, decreased cardiac apoptosis	[83]
31	Flavone	Chrysin	$C_{15}$ $H_{10}$ $O_{4}$	254.24	*Oroxylum indicum* (L.) Kurz, *Pinus monticola* Dougl., honey, etc.	STZ-induced diabetic rats	60	4 weeks	Inhibition of AGE-RAGE axis; activation of PPAR-γ	Anti-inflammatory, anti-oxidative stress	[84]
32	Biflavone	Kolaviron	$C_{31}$ $H_{24}$ $O_{12}$	588.5	*Garcinia kola* Heckel	STZ-induced diabetic rats	200	28 days	/	Anti-inflammatory, anti-oxidative stress	[85]
33	Flavonol	Galangin	$C_{15}$ $H_{10}$ $O_{5}$	270.24	*Alpinia officinarum* Hance	STZ-induced diabetic rats	15 mg/kg/d	6 weeks	/	Anti-inflammatory, anti-oxidative stress, decreased cardiac apoptosis, ameliorate dyslipidemia and myocardial injury, prevents DNA damage	[86]
34	Dihydroflavonol	Taxifolin	$C_{15}$ $H_{12}$ $O_{7}$	304.25	*Silybum marianum* (L.) Gaertn., *Tamarindus officinalis* Gaertn. and *Larix gmelinii* (Rupr.) Kuzen.	STZ-induced diabetic mice; HG-induced H9c2 cells	25, 50 and 100	4 weeks	/	Anti-oxidative stress, decreased cardiac apoptosis, improved diastolic dysfunction, ameliorated myocardium structure abnormality	[87]
35	Flavone	Wogonin	$C_{16}$ $H_{12}$ $O_{5}$	284.26	*Scutellaria baicalensis* Georgi	STZ-induced diabetic mice; HG-primary NRVMs	10; 10	16 weeks; 12 h	/	Anti-inflammatory, anti-oxidative stress, decreased myocardial fibrosis, decreased cardiac apoptosis	[88]
36	Chalcone	Hydroxysafflor yellow A	$C_{27}$ $H_{32}$ $O_{16}$	612.5	*Crocus sativus* L.	HFD + STZ-induced diabetic mice	60	12 weeks; 25 h	/	Anti-oxidative stress	[89]

AGE, advanced glycation end-product; AKT, protein kinase B; ARE , antioxidant response element; cGMP, cyclic guanosine monophosphate; EETs, epoxyeicosatrienoic acids; GSK-3β, glycogen synthase kinase 3β; HC, high cholesterol diet; HDAC4, histone deacetylase 4; HFD, high-fat diet; HG, hyperglycemia; HO-1, heme oxygenase-1; HS, high-sugar; IDH2, isocitrate dehydrogenase 2; IRS, insulin receptor substrate; JNK, c-Jun N-terminal protein kinase; Keap1, kelch-like ECH-associated protein 1; LV, left ventricular; MAPK, mitogen-activated protein kinases; MEF2, myocyte enhancer factor 2; miR-34a, microRNA-34a; MMPs, matrix metalloproteinases; mTOR, mammalian target of rapamycin; MyD88, myeloid differentiation factor 88; NFAT, nuclear factor of activated T cells; NF-κB, nuclear factor kappa-B; Nrf-2, nuclear factor erythroid 2-related factor 2; NOS, nitric oxide synthase; NRVMs, neonatal rat ventricular myocytes; PDE5, phosphodiesterase 5; PI3K, phosphoinositide 3-Kinase; PKB, protein kinase B; PKG, protein kinase G; PPARs, peroxisome proliferator-activated receptors; sGC, soluble guanylate cyclase; SIRT1, silent information regulator 1; SIRT3, silent information regulator 3; Smad, drosophila mothers against decapentaplegic protein; STZ, streptozotocin; TAC, transverse aortic constriction; TGF-β, transforming growth factor-β; tTG, tissue transglutaminase; ULK1, unc-51-like autophagy activating kinase 1; TLR4, toll-like receptor 4; DCM, diabetic cardiomyopathy; ROS, reactive oxygen species; ECM, extracellular matrix; ATP, adenosine triphosphate; KATP, ATP-sensitive K(+); RAGE, advanced glycosylation end-product receptor; SIRT5, silent information regulator 5.

### 3.1 Rutin (Troxerutin)

Rutin, as a natural flavonoid compound, is found naturally in common foods. It is especially abundant in *Ruta graveolens* L., *Scphora japonica *L., and so on. Rutin may represent a potential therapeutic agent for DM and its complications in the flavonoid family. As early as 1948, there were reports of rutin treating DM complications [90]. Rutin substantially improved cardiac function and structure in DCM, but the mechanism and effect are complex and multi-faceted.

Metabolic disorders, oxidative stress, and inflammatory reactions are involved in the occurrence and development of DCM, which interact to induce myocardial injury. The risk of oxidative stress and inflammatory responses is increased by metabolic disorders, and oxidative stress and chronic inflammation can lead to the development of metabolic diseases [91]. Studies have shown that rutin is effective in the treatment of DCM, through ameliorating myocardium metabolic abnormalities, oxidative stress, inflammation, and cellular apoptosis, in streptozotocin (STZ)-induced diabetic rats [22]. Inflammation and oxidative stress often complement and reinforce each other to form a vicious cycle. Rutin improves DCM by exerting both antioxidant stress and anti-inflammatory effects, and its cardioprotective effects were mediated by alterations in tumor necrosis factor α (TNF-α), C-reactive protein (CRP), and brain natriuretic peptide (BNP) levels [23]. ROS plays a role in the pathogeneses of myocardial repair/remodeling and myocardial dysfunction in DCM. Rutin has been shown to attenuate oxidative stress-induced myocardial remodeling and LV and myocardial dysfunction in DCM [24]. Another study reported something similar, therapeutic rutin administration reduced myocardial remodeling and improved myocardial function in vivo, at least in part by reducing oxidative damage and ectopic lipid deposition, inhibiting fibrosis, and promoting angiogenesis [25].

Alloxan is a synthetic pyrimidine derivative first synthesized in the 19th century, which causes necrosis by a selective, toxic effect in certain cells [92]. Fibrosis, the excess and unsuitable accumulation of extracellular matrix in various tissues, is a common occurrence in patients with advanced DM [93]. Rutin has been proven to be potential therapeutic target against alloxan-induced diabetic kidney disease (DKD) and DCM in experimental rats, through the prevention of metabolic acidosis and fibrosis [26]. The cardiovascular disease (CVD) and kidney disease are closely inter-related [94]. DKD is highly correlated with DCM in terms of consistency of initial etiology and similarity of underlying pathological mechanisms, which may be a key factor in the remarkable efficacy of natural products in treating both diseases simultaneously.Treating one disease while benefiting other diseases may also be the advantage of rutin, which has reference significance for the study of its role in diabetic complications.

Tissue transglutaminase (tTG) belongs to the transglutaminase family, members of which have a diverse array of enzymatic and non-enzymatic functions. The inhibition of tTG has been reported to benefit CVD, by decreasing myocardial fibrosis and reducing cardiomyocyte hypertrophy [95]. Rutin may inhibit the expression of tTG and regulate the progression of myocardial injury and fibrosis in STZ-induced DCM rats [27]. However, the pathway mechanism underlying the above processes remains unclear, as tTG has not been studied much in the context of DCM.

The ER is an organelle that is specialized in protein folding and trafficking. Through its stress and calcium handling, proteins co-operate to keep the myocardial cell properly functioning. Endoplasmic reticulum stress (ERS) serves an important role in the course of DCM’s pathological progression, which can cause cell dysfunction and apoptosis [96]. Rutin alleviated HG-induced myocardial cell dysfunction and apoptosis by inhibiting ERS [28].

Troxerutin, a derivative of the naturally occurring bioflavonoid rutin. The c-Jun N-terminal protein kinases (JNKs) form one sub-family of the mitogen-activated protein kinases (MAPK) group, mediate eukaryotic cell responses to a wide range of abiotic and biotic stress insults [97]. As a critical node for the insulin signal regulation mechanism, insulin receptor substrate (IRS) is essential for the prevention and treatment of DM. Insulin resistance has been linked to modifications in protein kinase B (PKB, also known as AKT) phosphorylation. Troxerutin appears to protect against DCM through inhibition of nuclear factor kappa B (NF-κB) and activation of the AKT/IRS/JNK signaling pathway [29].

### 3.2 Quercetin

Quercetin is one of the widely existing flavonoids, which is abundant in nature, and the quantity of quercetin in onion is the highest [98]. The cardioprotective function of quercetin seems to focus more on two aspects in the context of DCM: regulation of lipid metabolism disorder series reactions and intervention of abnormal cell death.

Abnormal energy metabolism plays a significant role in the occurrence and development of CVDs, and cardiac energy metabolism regulation is a new frontier in CVD treatment [99]. In the context of lipid metabolism disorder, the accumulation of lipid in the myocardium causes cardiac lipotoxicity and induces cardiac dysfunction. Quercetin attenuated cardiac diastolic dysfunction, up-regulated intracellular anti-oxidant stress mechanisms, prevented cardiac cholesterol accumulation, and decreased the increase in myocyte density resulting from high cholesterol [30]. In addition, quercetin may ameliorate cardiac dysfunction and fibrosis by reducing glycerophospholipid metabolism dysregulation [31]. A connection exists between lipid metabolism disorder and pathological myocardial hypertrophy. Quercetin ameliorated pro-hypertrophic signaling pathways regulating the hypertrophic response in the cardiomyocyte, which provoked the inhibition of pro-hypertrophic signals in Zucker Diabetic Fatty rats (fa/fa) [32].

Myocardial cell death is a crucial factor in the development and progression of different etiological cardiomyopathies [100]. Pyroptosis has been observed in different heart cell types in DCM, including cardiomyocytes, endothelial cells, and fibroblasts [101]. Quercetin inhibits the progression of cell pyroptosis, thereby alleviating DCM, and its mechanism of action is related to the activation of the nuclear factor erythroid 2-related factor 2 (Nrf-2) signaling pathway [33]. Myocardial apoptosis plays a vital role in the pathogenesis of CVD in the DM. Mitochondrial pathways of apoptosis are inhibited by Quercetin, which prevents the death of cardiomyocytes [34].

The silent information regulator 5 (SIRT5), as a representive of the Sirtuin family, is valued for its role in myocardial injury in diabetes. Quercetin may promote the desuccinylation of isocitrate dehydrogenase 2 (IDH2) through SIRT5, thus maintaining mitochondrial homeostasis, protecting cardiomyocytes from inflammatory conditions and improving myocardial fibrosis, and thus reduce the incidence of HF [35].

### 3.3 Naringenin

Naringenin is found mainly in citrus fruits (e.g., grapefruit) and others, such as tomatoes and cherries. Naringenin has emerged as an important natural phytochemical with potential for the treatment or prevention of various disorders, such as obesity, diabetes, cardiac diseases, and metabolic syndrome [102].

Cardiac hypertrophy is an adaptive response to stimulation, but pathological cardiac hypertrophy usually develops into HF. Naringenin improved cardiac hypertrophy *in vivo*, which may be related to up-regulation of the expression of cytochrome P450 2J3 (CYP2J3), elevated levels of epoxyeicosatrienoic acids (EETs), and the activation of peroxisome proliferator-activated receptors (PPARs) [36]. Cell experiments have also confirmed that EETs and PPARs function together, which may contribute to the anti-hypertrophic effect of naringenin *in vitro* under HG conditions [37]. EETs and PPARs seem to be effective signaling pathways for naringenin intervention in DCM, but relevant studies still need to be further enriched.

Naringenin can regulate the Nrf-2 and NF-κB classic signaling pathways to protect against diabetes-induced myocardial damage by reducing oxidative stress, inhibiting inflammation, fibrosis, and apoptosis [38]. This indicates that naringenin has a significant advantage in controlling pathological damage such as fibrosis and apoptosis.

### 3.4 Naringin

Naringin is a natural polyphenol bioflavonoid, is the same as Naringenin mainly found in citrus fruits. Naringin could significantly alleviate various physical and chemical stimuli induced cardiovascular disorders such as DCM, ischemic heart diseases, oxidative stress-induced cardiac injury and diet-induced cardiovascular dysfunctions [103].

NF-κB is one of the classic signaling pathways targeted to protect against diabetes-induced myocardial damage. Naringin protects cardiomyocytes against HG-induce dcardiac injury by up-regulating ATP-sensitive K(+) (KATP) channels and inhibiting the NF-κB signaling pathways [39].

The precise regulation of calcium homeostasis in cardiomyocytes is the key to maintaining the systolic function of the heart. Treatment of DCM with naringin protected cardiomyocytes by reducing diastolic ${\mathrm{Ca}}^{2+}$ overload, decreasing ROS production, and suppressing inflammation. In addition, naringin reduced the activity of calpain, increased cell viability, and restored the protein expression of Kir6.2, sulfonylurea receptor 1 (SUR1), and SUR2 sub-units of the KATP channels [40].

Furthermore, by mitigating mitochondrial oxidative stress-induced injuries and inhibiting the ERS-mediated apoptotic pathway, naringin may provide protection against diabetes-induced myocardial damage [41].

### 3.5 Icariin (Icariside II)

Icariin, a major flavonoid extracted from *Epimedium brevicornu* Maxim, has presented a wide range of pharmacological activities. icariin and icariside II (its bioactive form), have been found to have preventive and therapeutic effects on DCM in pre-clinical studies.

Mitochondrial dysfunction generates more ROS and disrupts the oxidative phosphorylation process which, in turn, leads to myocardial oxidative stress damage. Icariin’s cardioprotective effect against DCM is mediated by activation of the Apelin/Silent information regulator 3 (SIRT3) signaling pathway, which prevents mitochondrial dysfunction [42].

Icariin is a promising natural product in anti-fibrotic and myocardial amelioration. Transforming growth factor-β1 (TGF-β1) is regarded as a crucial mediator for tissue fibrosis, which causes tissue scarring by activating drosophila mothers against decapentaplegic protein (Smad) [104]. The cardiac functions restored by icariin can be achieved through inhibition of the TGF-β1/Smad pathway, and through the amelioration of ECM accumulation and myocardial fibrosis [43]. The ${\mathrm{Ca}}^{2+}$ homeostasis has implications for cardiac myocyte contraction and contributes to the manifestation of DCM. Icariin regulates ${\mathrm{Ca}}^{2+}$ homeostasis through nitric oxide synthase 3 (NOS3), phosphodiesterase 5A (PDE5A) and soluble guanylate cyclase (sGC)/cyclic guanosine monophosphate (cGMP)/protein kinase G (PKG) signaling pathways. Furthermore, ICA-induced inhibition of JUN and p65 ameliorated the irregular collagen metabolism and myocardial fibrosis [44].

Icariside II is the main pharmacological metabolite of icariin *in vivo*. Treatment with icariside II improved DCM through antioxidative stress, antiinflammatory, and anti-apoptotic effects. Thus, the above mechanism is mediated by the AKT/NOS/NF-κB signaling pathway [105].

### 3.6 Catechins ( {-}-Epigallocatechin-3-gallate, {-}-Epicatechin)

Catechins are a class of phenolic active compound extracted from edible plants such as tea. Standardized green tea extract, which is rich in catechins, prevented the initial myocardial damage in diabetic hearts from developing into DCM [45]. There are four main types of green tea catechins: (-)-epigallocatechin-3-gallate (EGCG), which accounts for approximately 60% of the green tea catechin content; (-)-epigallocatechin (EGC) (19%); (-)-epicatechin-3-gallate (ECG) (13.6%); and (-)-epicatechin (EC) (6.4%) [106].

EGCG is the major polyphenolic compound present in green tea, which attenuated cardiac dysfunction, reduced myocardial infarct size and myocardial fibrosis, and decreased apoptosis and oxidative stress by stimulating the silent information regulator 1 (SIRT1) signaling pathway [46]. AMP activated protein kinase (AMPK) is a highly conserved metabolic master regulator, mammalian target of rapamycin (mTOR) is a serine/threonine protease, and the AMPK/mTOR signaling pathway involved in both plays a leading role in the regulation of autophagy. EGCG attenuated myocardial fibrosis in DCM, and its underlying mechanisms were associated with activation of autophagy through supression of TGF-β/matrix metalloproteinases (MMPs) signaling pathway and modulation of AMPK/mTOR signaling pathway [47]. In addition, EGCG protected against cardiac injury through ameliorating the increase in metabolic risk factors, inflammation, oxidative stress, and apoptosis in DCM [48]. EGCG administration and autologous adipose-derived stem cells (ADSC) transplantation showed synergistically beneficial effects on DCM [107]. This may be due to the fact that EGCG reduces oxidative stress and restores of cardiac function when receiving ADSC [108].

Emerging evidence supports a beneficial action of the potential impact of EC on the development/progression of DCM. The cardiac fibroblasts cultured in HG acquired a profibrotic phenotype, which was blocked by EC. The underlying mechanism was likely mediated by the effects of the G-protein coupled estrogen receptor (GPER) on the Smad/TGF-β1 signaling pathway [49].

### 3.7 Scutellarin

Scutellarin is an herbal flavonoid glucuronide, extracted from *Scutellaria baicalensis* Georgi, with multiple pharmacological activities. Scutellarin has a series of effects such as anti-inflammation, anti-oxidative stress, improve heart function and inhibit myocardial fibrosis level. The specific mechanism includes inhibition of the activation of nucleotide-binding oligomerization domain-like receptor with a pyrin domain 3 (NLRP3) and NF-κB and activation of phospho-protein kinase B (p-AKT), Nrf-2, and heme oxygenase (HO-1) [50]. The activation of Toll-like receptor (TLR) signaling pathways is conducted through myeloid differentiation primary-response protein 88 (MyD88) and inhibitor-KB (IkB) kinases, which induce translocation of NF-κB into the nucleus to activate various inflammatory cytokines. The anti-oxidative stress effect of scutellarin depends on regulation of the TLR-4/MyD88/NF-κB signaling pathway [51]. Kelch-like ECH-associated protein 1 (Keap1) is an oxidative stress sensor, and Keap1 protein interactions with Nrf-2 are the main route for Nrf-2 activity regulation. Furthermore, modulation of the Nrf-2/Keap1 signaling pathway is the mechanism behind the anti-inflammatory properties of scutellarin [51].

Autophagy and apoptosis are often associated in the pathological process of DCM. Scutellarin can promote the autophagy signaling pathway by up-regulating autophagy-related factors (Beclin-1 and LC3-II) and inhibit the apoptotic signal pathway by down-regulating apoptosis-related factors (caspase-3, caspase-8, caspase-9, caspase-12, Bax, and Cyt-C), thereby relieving DCM [52]. The complex interplay between apoptosis and autophagy further inspired a treatment concept for CVD through balanced switching between the two responses [109]. However, the relationship between the intervention of scutellarin in apoptosis and autophagy has not yet been revealed.

### 3.8 Dihydromyricetin

Dihydromyricetin is an important plant flavonoid, extracted from vine tea (*Ampelopsis grossedentata* Hand-Mazz.), which has attracted great attention for its health-beneficial activities. Excessive or insufficient autophagy has been described as a contributing factor to many pathological conditions. Targeting specific microRNA (miRNA) for autophagy modulation may provide reliable promising therapeutic strategies for DCM. By reducing the expression of miR-34a, dihydromyricetin restores impaired autophagy and thus alleviates DCM [53]. Unc-51-like autophagy activating kinase 1 (ULK1)—one of the key elements of the autophagy activator complex—together with AMPK kinases, guarantee the precise function of autophagy. The autophagy regulation mechanism of dihydromyricetin is realized through AMPK/ULK1 signaling pathway activation [54].

Dihydromyricetin has the potential to be used in the treatment of DCM, as it reduced inflammation, anti-oxidative stress, improved cardiac dysfunction, ameliorated cardiac hypertrophy, inhibited myocardial fibrosis and suppressed necroptosis. The above effects were realized through SIRT3 signaling pathway activation [55].

### 3.9 Luteolin

Luteolin is a common flavonoid present in many types of plants, such as flowers, fruits, vegetables, medicinal herbs, and spices. Luteolin has displayed a wide range of pharmacological properties, including anti-oxidant, anti-microbial, anti-inflammatory, chemopreventive, chemotherapeutic, cardioprotective, anti-diabetic, and neuroprotective activities [110]. Luteolin has a significant pharmacological effect in terms of DCM prevention and treatment. It can significantly reduce the inflammatory phenotype and anti-oxidative stress, as well as preventing myocardial fibrosis, cardiac hypertrophy, and dysfunction. The mechanisms involved include activation of the Nrf-2 signaling pathway and inhibition of the NF-κB signaling pathway [56]. Cardiac remodeling is a major mechanism for the progression of HF in DCM. The process of cardiac remodeling is influenced by the increase in the activities of proteolytic enzymes [111]. Luteolin regulates AMPK and AKT/glycogen synthase kinase 3 (GSK-3) signaling pathways and reduces proteasome activity to alleviate cardiac hypertrophy [57].

HF is one of the pathological features of DCM and the final outcome of its development. The results of one study demonstrated that luteolin attenuates myocardial oxidation, thereby inhibiting the progression of LV dysfunction in mice model of HF [58].

### 3.10 Kaempferol

Kaempferol is a flavonoid aglycone found naturally in many plants, such as beans, bee pollen, broccoli, capers, cauliflower, cabbage, endive, fennel, and garlic [112]. Kaempferol acts as a potential therapeutic agent for the treatment of DCM, as it can prevent diabetes-induced inflammation, oxidative stress, myocardial fibrosis, and apoptosis, mechanically linked to the inhibition of NF-κB and Nrf-2 signaling pathway activation [59]. In addition, kaempferol attenuated DCM through the regulation of it in insulin and glucose effects, as well as a cardiac-independent mechanism that involves the activation of SIRT1 [60].

### 3.11 Genistein

Genistein is the natural isoflavone with a comprehensive range of pharmacological properties, such as anti-oxidant stress, anti-inflammatory, anti-bacterial, and anti-viral activities, as well as effects on diabetes and lipid metabolism [113]. Genistein improved the damage of diabetic myocardium by virtue of its anti-inflammatory and anti-oxidant effects. Its cardioprotective effect seems to be mediated by inhibiting the activities of TNF-α, CRP, and TGF-β1 [61]. Genistein can attenuate myocardial fibrosis in T1DM rats, where the underlying mechanisms may be associated to a reduction of serum creatine kinase MB isozyme (CK-MB), lactate dehydrogenase (LDH) leakage, and suppression of the TGFβ1/Smad3 signaling pathway [62].

### 3.12 Phloretin

Phloretin is one of the best-known and abundant dihydrochalcones, having significant pharmacological activity. SIRT1-mediated deacetylation has a significant impact on several biological processes, which include cellular senescence, apoptosis, glucose metabolism, lipid metabolism, oxidative stress, and inflammation [114]. Phloretin protected against HG-induced inflammation and fibrosis in H9c2 cell, by regulating the expression of SIRT1 [63]. In addition, phloretin acts as a promising natural agent through increased Nrf-2 expression and dissociation of the Keap1/Nrf-2 complex, suppressing HG-induced cardiomyocyte oxidation and fibrotic injury [64].

### 3.13 Silymarin

Silymarin is obtained from *Silybum marianum* (L.) Gaertn., which has principally been used over the centuries to treat liver disease. Studies have revealed other therapeutic effects of silymarin in terms of cardioprotection, neuroprotection, immune modulation, and cancer [115]. The therapeutic effect of silymarin on DCM has been newly discovered in recent years. Administration of silymarin attenuated myocardial fibrosis and collagen deposition through decreased p-Smad2/3 and TGF-β1 levels, and increased the level of Smad7 [65]. In addition, the treatment of diabetic subjects with silymarin may inhibit cardiomyocytes apoptosis, promote survival and restoration of pancreatic β-cells [66].

### 3.14 Fisetin

Fisetin is a flavonoid with significant biological activity, which is found in many fruits and vegetables such as strawberries, persimmons, apples, onions, grapes, and cucumbers. Fisetin might be worth considering the therapeutic potential of fisetin for human DCM, which attenuates the development of DCM by ameliorating oxidative stress, inflammation, and apoptosis [67]. Protein kinase R (PKR) is a key inducer of inflammation, oxidative stress, insulin resistance, and glucose homeostasis in DM. Fisetin can preserve cardiac function and prevent further cardiac damage in diabetes through anti-inflammatory, improving cardiac glucose metabolism, suppression of FAs oxidation, anti-fibrotic, and anti-apoptotic effects. The above role may be related to suppression of PKR [68].

### 3.15 Puerarin

Puerarin is the most important phytoestrogen extracted from *Pueraria montana var. lobata* (Ohwi) Maesen & S. M. Almeida, and is widely used as a clinical auxiliary drug for the treatment of metabolic disorders and CVD. Puerarin may have promising therapeutic potential for DCM, with related to the attenuation of inflammation and fibrotic. Further evidence comes from the result that puerarin significantly inhibited the production of pro-inflammatory cytokines by blocking NF-κB signaling pathways [69]. Puerarin-V, a new form of puerarin, positively improved DCM by improving mitochondrial respiration, suppressing myocardial inflammation, inhibiting pyroptosis, and maintaining the structural integrity of the myocardium [70].

### 3.16 Aspalathin

Aspalathin is abundantly present in *Aspalathus lineari*, a plant from South African often used as a herbal tea. It increases glucose oxidation and modulates fatty acid utilization, producing a favorable substrate shift in H9c2 cells. Such a favorable shift may be of importance in the protection of the myocardium against cell apoptosis [71]. Related mechanisms include maintaining cellular homeostasis, modulating anti-inflammatory and anti-oxidative stress, and protecting the myocardium against HG-induced apoptosis through activation of Nrf-2 [72].

### 3.17 Liquiritin (Liquiritigenin, Isoliquiritigenin)

Liquiritigenin, liquiritin, and isoliquiritigenin are natural flavonoids distributed in *Glycyrrhizae Radix et* Rhizoma, which has been widely used as a herbal medicine for centuries in China. Liquiritin may be a promising candidate for the treatment of diabetes-related myocardial fibrosis, which had a protective effect against myocardial fibrosis through the suppression of NF-κB and MAPKs signaling pathways [73]. Liquiritigenin suppress myocardial fibrosis and inflammation, by inactivating the NF-κB signaling pathway [74]. Like the first two flavonoids, isoliquiritigenin has high research value in DCM. Isoliquiritigenin has anti-inflammatory, anti-oxidative stress, inhibits fibrosis, and restrain apoptosis in DCM. The mechanism underlying this protective effect has been implicated as involving the inhibition of MAPKs and induction of the Nrf-2 signaling pathway [75].

### 3.18 Others

Daidzein is an isoflavone extract from soy, and the role of it in diabetic cardiac complications has been well studied and proved. Daidzein has therapeutic potential against diabetes-related cardiac complications, which may reduce glucotoxicity-induced cardiac mechanical dysfunction [116]. Daidzein prevented the progression of DCM through an anti-oxidative mechanism by inhibiting the activation of NADPH oxidase 4 (NOX4) in cardiomyocytes. It also improved the AMPK and SIRT1 signaling pathway and prevented changes in the structure and function of the myocardium [76].

Apigenin is a natural flavonoid found in many dietary plant foods. Apigenin have been reported to be beneficial a variety of CVD, such as atherosclerosis, hypertension, ischemia/reperfusion-induced myocardial injury, DCM, and drug-induced cardiotoxicity [117]. Apigenin effectively mitigated diabetes-induced myocardial inflammation, oxidative stress, fibrosis, and apoptosis, both *in vivo* and *in vitro*. The internal mechanism is that apigenin suppresses the phosphorylation of the NF-κB inhibitor IkB-α and translocation of NF-κB/P65, while suppressing the expression of TNF-α [77].

Myricitrin is a member of the flavonol class of flavonoids, which is commonly derived from vegetables, fruits. Myricitrin exerts cardioprotective effects against DCM through the anti-inflammatory, anti-oxidative stress and inhibition of apoptosis. Its mechanism of action is through attenuating the Nrf-2 inhibition in DCM, by the regulation of AKT and extracellular signal-regulated kinase (ERK) phosphorylation [78].

Nobiletin is a polymethoxyflavone primarily present in citrus fruits. Nobiletin mitigates cardiac dysfunction and interstitial fibrosis in DCM. These effects of nobiletin may be attributed to the suppression of JNK, P38, and NF-κB [79].

Myricetin is a hexahydroxyflavone and isolated from the leaves of *Morella rubra* Lour. Myricetin possesses potential protective effects in DCM, which attributed to alleviate oxidative stress, inflammation, apoptosis, and fibrosis.The underlying mechanisms of it at least partly associated to the inhibition of the IκB-α/NF-κB/p65 and TGF-β/Smad signaling pathways and enhancing the expression of Nrf-2 [80].

Baicalein is a trihydroxyflavone derived from the roots of *Scutellaria baicalensis* Georgi. Baicalein was effective in preventing damage to DCM caused by oxidative stress and inflammation, and the PI3K/AKT signaling pathway may have been involved in mediating these effects [81].

Sciadopitysin is an amentoflavone-type biflavonoid contained in *Taxus chinensis*, which exerts anti-inflammatory and anti-oxidative effects. Sciadopitysin alleviated HG-caused oxidative stress and apoptosis in cardiomyocytes by activating the PI3K/PKB/GSK-3β signaling pathway [82].

Spiraeoside, also known as quercetin-4-O-β-D-glucoside, is mainly derived from *Spiraea salicifolia* L., and protected cardiomyocytes from HG-induced oxidative stress, cell injury, and apoptosis through activation of the PI3K/Akt/Nrf-2 signaling pathway [83].

Chrysin is a dihydroxyflavone which occurs naturally in many plants, honey, and propolis. The binding of AGE to its receptor AGE (RAGE) enhances oxidative stress, thereby causing damage to cells and tissues. Chrysin significantly ameliorated isoproterenol-induced myocardial injury through anti-inflammatory and anti-oxidative stress. The PPAR-γ activation and inhibition of AGE-RAGE-mediated above chrysin’s effect [84].

Kolaviron, an important component of the seed of *Garcinia kola* Heckel, possesses a variety of biological activities, including anti-inflammatory and anti-oxidant stress properties. Kolaviron attenuated oxidative and inflammation cardiovascular injury in DCM [85].

Galangin is a naturally occurring flavonol glycoside found in *Alpinia officinarum* Hance. Galangin ameliorated HG, hyperlipidemia, oxidative stress, inflammation and apoptosis, and prevented myocardial damage in DCM [86].

Taxifolin is a dihydroflavonol commonly found in onion, *Silybum marianum *(L.) Gaertn., *Tamarindus officinalis* Gaertn., and *Larix gmelinii* (Rupr.) Kuzen. Taxifolin exerted cardioprotective effects against DCM by anti-oxidant stress and inhibition of apoptosis [87].

Wogonin is a flavonoid acting as a yellow color pigment, obtained from the roots of the plant *Scutellaria Baicalensis* Georgi. The anti-apoptotic, anti-inflammatory, anti-fibrosis, and anti-oxidative stress bioactivities of wogonin are expected to alleviate the progression of DCM [88].

Hydroxysafflor yellow A is the main bioactive compound of a traditional Chinese medicine (TCM) obtained from *Crocus sativus* L. Research has shown that the pharmacokinetics of Hydroxysafflor yellow A changed significantly in DCM, which may improve the anti-oxidative stress effect of the drug [89].

## 4. Terpenoids

**Fig. 3.**
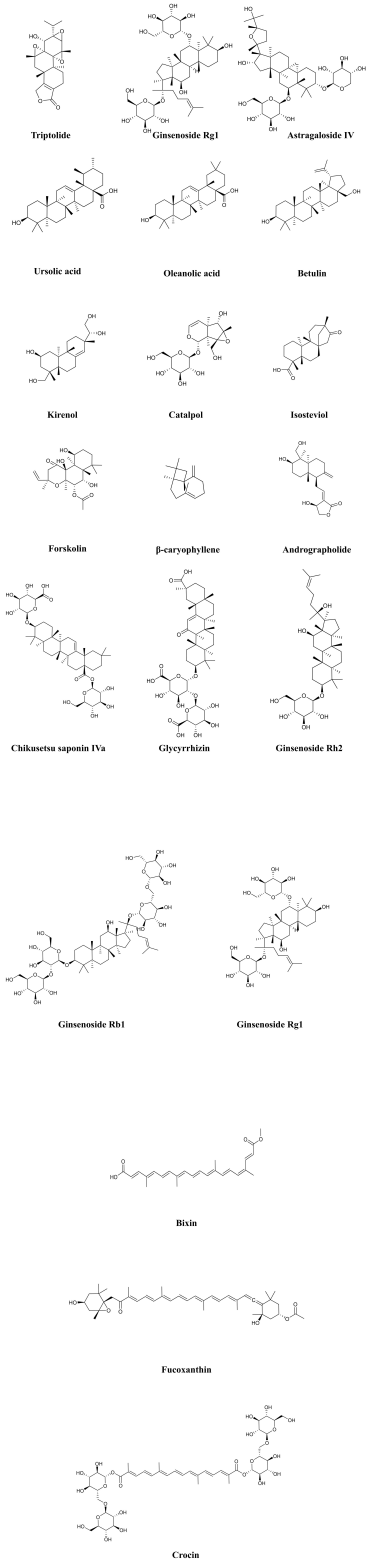
**Chemical structures of considered terpenoids**. Note: Due to the complex chemical structure of terpenoids, we have rearranged the order.

Terpenoids are the largest and most diverse group of natural products, attracting extensive attention due to their various biological activities [118, 119]. Terpenoids, which are composed of five carbon isoprene units, are classified into various subclasses based on their distinct chemical structures, including hemiterpenoids, monoterpenoids, sesquiterpenoids, diterpenoids, sesterterpenoids, triterpenoids, and tetraterpenoids. Terpenoids have been widely used in the treatment of numerous diseases because of their extensive range of biological activities, including their anti-microbial, anti-cancer, hypotensive, anti-hyperlipidemic, anti-hyperglycemic, anti-inflammatory, anti-oxidant, anti-parasitic, immunomodulatory, and anti-cholinesterase activities [120]. The significant pharmacological effects of terpenoids have been further demonstrated in DCM studies. A total of 19 terpenoids had effective therapeutic intervention effects on DCM, including 1 iridoid, 1 sesquiterpenoid, 6 diterpenoids, 9 triterpenoids, and 2 tetraterpenoids. Table 2 (Ref. [121, 122, 123, 124, 125, 126, 127, 128, 129, 130, 131, 132, 133, 134, 135, 136, 137, 138, 139, 140, 141, 142, 143, 144, 145, 146, 147, 148, 149, 150]) provides the basic information and mechanisms of these 19 terpenoids from recent studies on DCM, while Fig. 3 presents the chemical structures of the 19 terpenoids.

**Table 2.** **Basic information and mechanisms of 19 terpenoids from recent studies on DCM**.

Number	Terpenoid Subclass	Compounds	Molecular formula	Weight (g/mol)	Resources	Animal/Cell model	Dosage (mg/kg/d; µm)	Dosing cycle	Target/Pathways/Mechanism	Effects	Reference
1	Diterpenoid	Triptolide	$C_{20}$ $H_{24}$ $O_{6}$	360.40	*Tripterygium wilfordii* Hook. f.	STZ-induced diabetic rats	100, 200, and 400 (µg/kg/d)	6 weeks	Up-regulated MAPK signaling pathway	Improved myocardial energy metabolism	[121]
						STZ-induced diabetic rats; HG-induced H9c2 cells	100, 200, and 400 (µg/kg/d); 20 (ng/mL)	6 weeks; 48 h	Suppression of NF-κB signaling pathway	Anti-inflammatory, decreased myocardial fibrosis	[122]
						HG and high-fat + STZ-induced diabetic rats	50, 100, 200 µg/kg/d	8 weeks	Inhibition of TLR4-induced NF-κB/IL-1β signaling pathway, suppression of NF-κB/TNF-α/VCAM-1 signaling pathway and down-regulation of TGF-β1/α-SMA/Vimentin signaling pathway	Regulated immune system, anti-inflammatory, decreased myocardial fibrosis, improved left ventricle function	[123]
2	Triterpenoid	Ginsenoside-Rb1	$C_{54}$ $H_{92}$ $O_{23}$	1109.30	*Panax ginseng* C. A. Mey.	HFD + STZ-induced diabetic mice	40	8 weeks	/	Improved calcium signaling	[124]
						Diabetic db/db mice	25, 50, and 100	12 weeks	Regulated adipocytokine pathway	Anti-inflammatory, decreased myocardial fibrosis, ameliorated apoptotic, anti-oxidative stress, reduced cardiac hypertrophy	[125]
3	Triterpenoid	Ginsenoside-Rg1	$C_{42}$ $H_{72}$ $O_{14}$	801.00	*Panax ginseng* C. A. Mey.	HFD + STZ-induced diabetic rats	10, 15, and 20	12 weeks	/	Decreased myocardial apoptosis, ameliorated oxidative stress	[126]
						HFD + STZ-induced diabetic rats	10, 15, and 20	12 weeks	/	Decreased myocardial apoptosis, reduced ER stress	[127]
4	Triterpenoid	Ginsenoside Rh2	$C_{36}$ $H_{62}$ $O_{8}$	622.90	*Panax ginseng* C. A. Mey.	STZ-induced diabetic rats; HG-induced H9c2 cells	5; 50	4 weeks; 24 h	Suppression of PPARδ/STAT3 Signaling pathway	Decreased myocardial fibrosis	[128]
5	Triterpenoid	Astragaloside IV	$C_{41}$ $H_{68}$ $O_{14}$	785.00	*Astragalus membranaceus var. mongholicus* (Bunge) P. K. Hsiao	HFD + STZ-induced diabetic rats	80	8 weeks	/	Anti-inflammatory, decreased myocardial fibrosis, improved lipid metabolism	[129]
						HG-induced H9c2 cells	25, 50, and 100	24 h	Regulated miR-34a/Bcl2/(LC3II/LC3I) and pAKT/Bcl2/(LC3II/LC3I) pathway	Anti-oxidative stress, inhibition autophagy	[130]
						STZ-induced diabetic rats; HG-induced H9c2 cells	10, 20, and 40; 20, 40, and 80	16 weeks; 24 h	Regulated PGC-1α and Nrf-1	Regulate energy metabolism	[131]
6	Diterpenoid	Crocin	$C_{44}$ $H_{64}$ $O_{24}$	977.00	*Crocus sativus* L.	HFD + STZ-induced diabetic rats; HG-induced adult rat cardiac myocytes	10 and 20; 1 and 10 (mmol)	2 weeks; 3 h	/	Decreased myocardial apoptosis, inhibition autophagy	[132]
						HFD + STZ-induced diabetic rats	50	8 weeks	/	Anti-oxidative stress	[133]
						STZ-induced diabetic rats	40	4 weeks	Activation of PPARγ	Anti-inflammatory, anti-oxidative stress	[134]
7	Triterpenoid	Ursolic acid	$C_{30}$ $H_{48}$ $O_{3}$	456.70	*Arctostaphylos uva-ursi (L.) Spreng., Prunella vulgaris* L., *Ilex rotunda* Thunb., etc.	STZ-induced diabetic rats	35	8 weeks	/	Anti-inflammatory, anti-oxidative stress, decreased myocardial fibrosis	[135]
						HFD + STZ-induced diabetic mice	100	8 weeks	/	Anti-inflammatory	[136]
8	Triterpenoid	Glycyrrhizin	$C_{42}$ $H_{62}$ $O_{16}$	822.90	*Glycyrrhiza uralensis* Fisch.	ZDF rats; HG-induced AC16 human CMs cell	50; 50	4 weeks; 24 h	Activation of Nrf-2 signaling pathway, inhibition of CXCR4/SDF1 and TGF-β/p38MAPK signaling pathway	Anti-inflammatory, anti-oxidative stress, decreased myocardial fibrosis	[137]
						HS + HFD-induced diabetic mice	150	8 weeks	Inhibition of HMGB1	Anti-inflammatory	[138]
9	Tetraterpenoid	Fucoxanthin	$C_{42}$ $H_{58}$ $O_{6}$	658.90	Brown seaweed	STZ-induced diabetic rats; HG-induced H9c2 cells	200; 1	12 weeks; 48 h	Regulation of BNIP3/Nix and Nrf-2 signaling pathway	Decreased myocardial fibrosis, reduced cardiac hypertrophy, reversed morphological and functional abnormalities of mitochondria, improved mitophagy	[139]
						STZ-induced diabetic rats; HG-induced H9c2 cells	200 and 230; 1	12 weeks; 48 h	Regulation of Nrf-2 signaling pathway	Decreased myocardial fibrosis and hypertrophy, anti-oxidative stress	[140]
10	Triterpenoid	Oleanolic acid	$C_{30}$ $H_{48}$ $O_{3}$	456.70	*Canarium oleosum* (Lam.) Engl., etc.	STZ-induced diabetic rats	80/2 d	2 weeks	Regulation of HO-1/Nrf-2 signaling pathway and GS/GP signaling pathways	Anti-oxidative stress	[141]
11	Triterpenoid	Chikusetsu saponin IVa	$C_{42}$ $H_{66}$ $O_{14}$	795.00	*Swartzia simplex* (Sw.) Spreng., *Anredera baselloides* (Kunth) Baill.	HG-induced H9c2 cells and rat primary cardiomyocytes	12.5, 25 and 50	24 h	Activation of SIRT1/ERK1/2 and Homer1a signaling pathway	Anti-oxidative stress, decreased myocardial apoptosis, ameliorated ${\mathrm{Ca}}^{2+}$ accumulation	[142]
12	Triterpenoid	Betulin	$C_{30}$ $H_{50}$ $O_{2}$	442.70	*Betula platyphylla* Sukaczev	Diabetic db/db mice; HG-induced H9c2 cells	20 and 40; 30 (mmol/L)	12 weeks; 24 h	Reversed the SIRT1/NLRP3/NF-κB signaling pathway	Anti-inflammatory, improved insulin resistance and hyperglycemia	[143]
13	Diterpenoid	Kirenol	$C_{20}$ $H_{34}$ $O_{4}$	338.50	*Sigesbeckia orientalis* L., *Sigesbeckia glabrescens* Makino, *Sigesbeckia pubescens* Makino, etc.	The GK rat; HG‐induced CMs and CFs from rats	0.5 and 2; 20 and 40	8 weeks; 12, 24, or 36 h	Suppression of NF-κB, MAPK and TGF‐β/Smad signaling pathways	Anti-inflammatory, decreased myocardial fibrosis, ameliorated apoptosis	[144]
14	Iridoid	Catalpol	$C_{15}$ $H_{22}$ $O_{10}$	362.33	*Rehmannia glutinosa* (Gaertn.) Libosch. ex Fisch. & C. A. Mey.	HS + HFD + STZ-induced diabetic rats; HG-induced Mouse cardiomyocytes	10; 1, 2, and 4 (mg/mL)	12 weeks; 24 h	Regulated Neat1/miR-140-5p/HDAC4 axis	Decreased myocardial apoptosis	[145]
15	Diterpenoid	Isosteviol	$C_{20}$ $H_{30}$ $O_{3}$	318.40	*Stevia rebaudiana* (Bertoni) Bertoni	STZ-induced diabetic rats	8	11 weeks	Inhibition of ERK and NF-κB signaling pathways	Anti-inflammatory, anti-oxidative stress	[146]
16	Tetraterpenoid	Bixin	$C_{25}$ $H_{30}$ $O_{4}$	394.50	*Bixa orellana* L.	HFD-induced diabetic rats; HG-induced H9c2 cells	50, 100, and 200; 20, 40, and 80	14 weeks; 24 h	Activation of Nrf-2 signaling pathway	Anti-inflammatory, anti-oxidative stress, decreased myocardial fibrosis	[147]
17	Sesquiterpenoid	β-caryophyllene	$C_{15}$ $H_{24}$	204.35	*Citrus* ×*limon* (Linnaeus) Osbeck, *Cinnamomum cassia* (L.) D. Don, *Piper nigrum* L., etc.	STZ-induced diabetic rats	100 and 200	4 weeks	Inhibition of NF-κB signaling pathways	Anti-inflammatory, anti-oxidative stress, decreased myocardial fibrosis	[148]
18	Diterpenoid	Andrographolide	$C_{20}$ $H_{30}$ $O_{5}$	350.40	*Andrographis paniculata* (Burm. f.) Wall. ex Nees in Wallich	STZ-induced diabetic mice; HG-induced H9c2 cells	1, 10, and 20; 1, 5 and 10 (µM)	12 weeks; 48 h	Suppression of NF-κB and NOX/Nrf-2 signaling pathway	Anti-inflammatory, anti-oxidative stress, decreased myocardial apoptosis	[149]
19	Diterpenoid	Forskolin	$C_{22}$ $H_{34}$ $O_{7}$	410.50	*Coleus forskohlii* (Willd.) Briq.	STZ-induced diabetic mice	2	4 weeks	/	Decreased myocardial fibrosis, anti-oxidative stress	[150]

α-SMA, α-smooth muscle actin; Bcl2, B-cell lymphoma-2; BNIP3, BCL2 interacting protein 3; CFs, cardiofibroblasts; CMs, cardiomyocyte; CXCR4, C-X-C chemokine receptor type 4; ERK1/2 , extracellular signal-regulated kinase1/2; GK, Goto–Kakizaki; GP, glycogen phosphorylase; GS, glycogen synthase; HDAC4, Histone deacetylase 4; HO-1, heme oxygenase-1; LC-3, light chain 3; IL-1β, Interleukin-1β; MAPK, mitogen-activated protein kinases; miR-140-5p, microRNA-140-5p; miR-34a, microRNA-34a; Neat1, long non-coding RNA nuclear paraspeckle assembly transcript 1; NF-κB, nuclear factor kappa-B; Nix, NIP3-like protein X; NLRP3, nucleotide-binding oligomerization domain-like receptor with a pyrin domain 3; NOX, NADPH oxidase; Nrf-1, nuclear respiratory factor-1; Nrf-2, nuclear respiratory factor-2; PGC1α, peroxisome proliferator-activated receptor-γ coactivator1α; PPARδ, peroxisome proliferator-activated receptor δ; PPARγ, peroxisome proliferator-activated receptor γ; SDF1, stromal cell-derived factor-1; SIRT1, silent information regulator 1; STAT3, signal transducer and activator of transcription 3; STZ, streptozotocin; TGF-β, transforming growth factor-β; TLR4, toll-like receptor 4; TNF-α, tumor necrosis factor α; VCAM-1, vascular cell adhesion molecule 1; DCM, diabetic cardiomyopathy; HG, hyperglycemia; HFD, high-fat diet; HMGB1, high-mobility group box-1.

### 4.1 Triptolide

Traditional herbal medicine (THM) provides a fertile ground for modern drug development, and triptolide is one of the “poster children” that exemplifies the potential and promise of transforming THM into modern drugs [151].

The loss of metabolic flexibility leads to a decrease in the utilization of cardiac matrix and the efficiency of ATP production in DM patients. Triptolide therapy improved cardiac function and increased cardiac energy metabolism, through up-regulation of MAPK signaling transduction *in vivo* [121]. The activation of NF-κB induces the production of a large number of pro-inflammatory cytokines and induces inflammation. Triptolide therapy significantly reduced cardiac inflammation and fibrosis by inhibiting the activity and expression of NF-κB, ultimately leading to improved LV dysfunction [122]. The protective effect of triptolide on DCM is diverse and its mechanism is complex. The protective effects of triptolide against DCM might be attributed to inhibition of the TLR-4-induced NF-κB/Interleukin-1β (IL-1β) signaling pathway, down-regulation of the TGF-β1/α-smooth muscle actin (α-SMA)/Vimentin fibrosis signaling pathway, and suppression of the NF-κB/TNF-α/vascular cell adhesion molecule 1 (VCAM-1) inflammatory signaling pathway [123].

### 4.2 Ginsenoside (Ginsenoside Rb1, Ginsenoside Rg1 and Ginsenoside Rh2)

Ginsenosides derived from the roots and rhizomes of *Panax ginseng* C. A. Meyer, have been utilized as an adjuvant treatment for DM in China. Ginsenosides can provide myocardial protection in DM through anti-oxidant stress, improved cardiac function, attenuated myocardial fibrosis, and reduced apoptosis [152].

Ginsenoside Rb1 is the most abundant triterpenoid saponin, which belongs to ginsenoside type A. One study has suggested that ginsenoside Rb1 could serve as a viable adjunctive therapeutic agent for DCM. The activity of RyR2 and SERCA 2a was regulated by Ginsenoside Rb1, which improved calcium signaling [124]. Adipocytokines are secreted from adipose tissue, which play critical roles in diabetes and obesity. Ginsenoside Rb1 reduced lipid levels through apoptocytokine signaling and reduced oxidative stress, hypertrophy, inflammation, fibrosis, and apoptosis in DCM [125].

Ginsenoside Rg1, which belongs to ginsentriol type B, has significant myocardial protective effect of DCM and its efficacy was associated with reduced oxidative stress and attenuated myocardial apoptosis [126]. Ginsenoside Rg1 treatment attenuated diabetic myocardial damage in DCM by reducing ERS-induced apoptosis [127].

Ginsenoside Rh2, which belongs to the ginsenodiol saponins, is suitable for the development of an alternative remedy for myocardial fibrosis. Research has indicated its effectiveness in improving cardiac function and fibrosis, through increasing PPARδ signaling pathway [128].

### 4.3 Astragaloside IV

Astragaloside IV (AS-IV), one of the main compounds from *Astragalus membranaceus var. mongholicus* (Bunge) P. K. Hsiao, is a cycloartane-type triterpene glycoside chemical.

The changes in metabolic pattern affect the cardiac remodeling and functional change. Astragaloside IV can prevent myocardial injury caused by T2DM, and its mechanism may involve improving myocardial lipid metabolism [129]. In addition, astragaloside IV can regulate the release of peroxisome proliferator-activated receptor-γ coactivator-1α (PGC-1α) and nuclear respiratory factor-1 (Nrf-1) to rescue the abnormal myocardial mitochondrial energy metabolism, thus decreasing the myocardial damage in DCM [130].

The light chain 3 (LC-3) plays an important role in autophagy and is used as a molecular marker of autophagy. B-cell lymphoma-2 (Bcl2), a protein that also plays a significant role in autophagy, interacts with a variety of co-factors to trigger a cascade of autophagy proteins. Astragaloside IV inhibits HG-induced oxidative stress and autophagy, through the miR-34a/Bcl2/(LC3II/LC3I) and phosphorylated-Serine473-AKT (pAKT)/Bcl2/(LC3II/LC3I) pathways *in vitro* [131].

### 4.4 Crocin

Crocin, an abundant anti-oxidant ingredient of *Crocus sativus* L. (saffron), exhibits significant protective effects against myocardial injury, especially in DCM. Crocin enhances cardiac dysfunction by restoring autophagy and preventing apoptosis in DCM [132]. Crocin resulted in a higher increase of anti-oxidant levels and a more reduced lipid peroxidation rate (malondialdehyde (MDA) content) in the heart of T2DM, and it was also revealed that a combination of crocin with voluntary exercise was more effective than crocin therapy alone [133].

Crocin, as an anti-oxidant compound, protects the myocardium against diabetes complications through activation of PPARγ, elevating anti-oxidant capacity, decreasing inflammatory cytokines, and reducing of cardiac injury marker activities [134]. In addition, there are studies that have reflected the potential involvement of the PPARγ signaling pathway in the protective effects of crocin in DCM [153].

### 4.5 Ursolic Acid

Ursolic acid is a natural pentacyclic triterpenoid, which possesses diverse pharmacological actions. Ursolic acid is capable of improving the cardiac structure and function *in vivo* by attenuating oxidative stress, inflammation, and fibrosis [135]. In addition, ursolic acid had an obvious protective effect on myocardial injury in DCM, and its mechanism may be associated with the inhibition of NLRP3 inflammasome activation, reduced IL-1β generation, and the alleviation of myocardial injury [136].

### 4.6 Glycyrrhizin

Glycyrrhizin, also called glycyrrhizic acid, is a triterpenoid saponin mainly isolated from *Glycyrrhiza uralensis* Fisch. Glycyrrhizin has presented cardioprotective effects in diabetic cardiac atrophy, which may be mediated through activation of Nrf-2 and inhibition of C-X-C chemokine receptor type 4 (CXCR4)/stromal cell-derived factor-1 (SDF1) as well as the TGF-β/p38MAPK signaling pathway [137]. In addition, glycyrrhizin prevents cardiac inflammation and decelerates the development of DCM by antagonizing extracellular high-mobility group box-1 (HMGB1) [138].

### 4.7 Fucoxanthin

Fucoxanthin, as the natural product of carotenoids, can potentially be obtained from marine algae. The NIP3-like protein X (Nix) is a key protein for mitophagy during the maturation of reticulocytes. Fucoxanthin reduced the accumulation of TGF-β1, fibronectin and α-SMA to relieve myocardial fibrosis *in vivo*. Fucoxanthin up-regulated Bcl2 interacting protein 3 (BNIP3)/Nix to promote mitophagy and enhanced Nrf-2 signaling pathway to alleviate oxidative stress, thereby inhibiting hypertrophy *in vitro* [139]. In addition, fucoxanthin can regulate Nrf-2/Keap1 signaling pathway to reduce myocardial hypertrophy in DCM [140].

### 4.8 Others

Oleanolic acid is a naturally occurring pentacyclic triterpenoid that is widely distributed in plants. Treatment with oleanolic acid blunted HG-induced oxidative stress, apoptosis, and the ubiquitin–proteasome system in heart cells [154]. In recent years, the value of oleanolic acid in the field of DCM has been gradually explored, and its protective effect against cardiac injury caused by oxidative stress has been revealed. Glycogen synthase (GS) and glycogen phosphorylase (GP) are two key enzymes for glycogen synthesis and metabolism. Oleanolic acid protects against DCM, through the HO-1/Nrf-2 and GS/GP signaling pathways [141].

Chikusetsusaponin IVa is a natural product found in *Swartzia simplex* (Sw.) Spreng., *Anredera baselloides* (Kunth) Baill., and other plants. Chikusetsusaponin IVa protected cardiomyocytes from HG-triggered oxidative stress and calcium overload. The underlying mechanisms of Chikusetsusaponin IVa-mediated cardioprotection might be attributable to the regulation SIRT1/ERK1/2/Homer1a signaling pathways [142].

Betulin is a natural triterpenoid product contained in several medicinal plants, including *Betula platyphylla* Sukaczev. Betulin significantly protected against DCM by effectively improving insulin resistance, HG, and inflammation. Research has shown that Betulin plays the heart-protective role described above by regulating the SIRT1/NLRP/NF-κB signaling pathway [143].

Kirenol is an ent-pimarane-type diterpenoid that has been reported from *Sigesbeckia orientalis* L., *Sigesbeckia glabrescens* Makino, *Sigesbeckia pubescens* Makino, and others. The Goto–Kakizaki (GK) rat is a non-obese, non-hypertensive model of T2DM, like humans, it has a susceptibility locus on chromosome 10. The cardioprotective effect of kirenol in GK rats is mediated by regulation of the NF-κB, MAPK, and TGF-β/Smad signaling pathways [144].

Catalpol is an iridoid glycoside extracted from the roots of *Rehmannia glutinosa* (Gaertn.) Libosch. ex Fisch. & C. A. Mey. Catalpol might stimulate the Neat1/miR-140-5p/Histone deacetylase 4 (HDAC4) signaling pathways, thereby leading to inhibition of HG-induced myocardial apoptosis [145].

Isosteviol, an ent-beyerane diterpenoid found in *Stevia rebaudiana* (Bertoni) Bertoni, has been repeatedly reported as possessing potent cardioprotective activity. Isosteviol sodium (STVNa) is an improved formulation with higher solubility and bioavailability, which therapeutic effect is achieved by reducing oxidative stress and inflammation in DCM. The mechanism is based on inhibiting ERK and NF-κB signaling pathways [146].

Bixin, a natural carotenoid extracted from *Bixa orellana* L., possesses anti-oxidant stress and anti-inflammatory effects. Bixin might be a novel and protective agent with therapeutic activity against DCM which acts by suppressing fibrosis, anti-inflammatory and anti-oxidative stress. Its related intervention mechanism is mediated by Nrf-2 signaling pathway activation [147].

β-caryophyllene is widely found in *Citrus* × *limon* (Linnaeus) Osbeck, *Cinnamomum cassia* (L.) D. Don, *Piper nigrum* L., etc. The combination of β-caryophyllene with L-arginine improved cardiac functions by attenuating inflammation through NF-κB signaling pathway inhibition in DCM [148].

Andrographolide is a labdane diterpenoid that is produced by the plant *Andrographis paniculata*. Andrographolide treatment exerts cardioprotective effects through modulation of the NADPH oxidase (NOX)/Nrf-2 signaling pathway. The therapeutic potential of andrographolide in the treatment of DCM is demonstrated by its ability to attenuation oxidative stress, inflammation, and apoptosis [149].

Forskolin is a labdane diterpenoid isolated from the Indian Coleus plant *Coleus forskohlii* (Willd.) Briq. Forskolin treatment in DCM significantly blocked oxidative stress and reduced myocardial fibrosis [150]. However, the specific signaling pathway mechanism underlying the role of forskolin remains to be elucidated.

## 5. Alkaloids

**Fig. 4.**
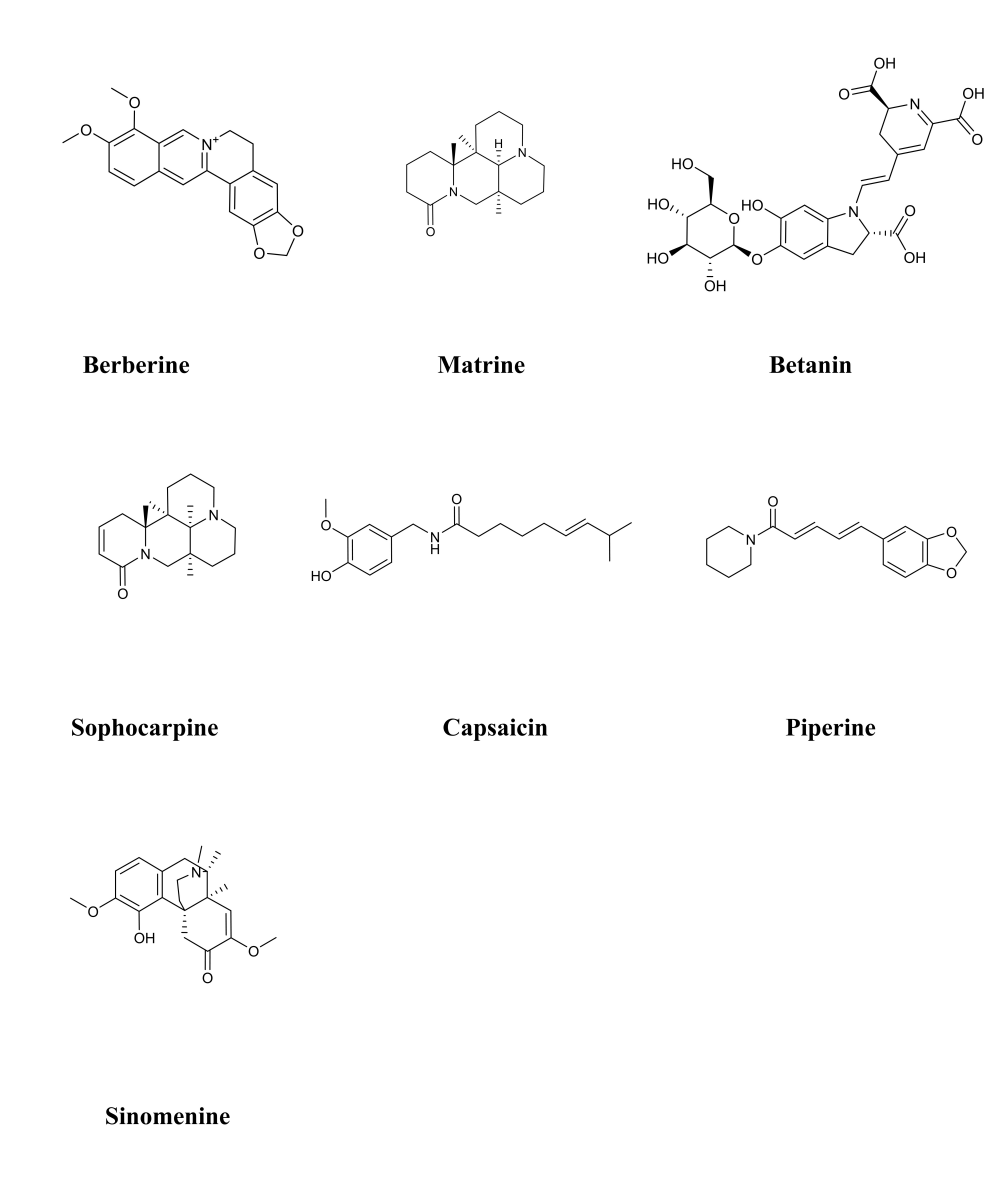
**Chemical structures of considered alkaloids**.

Alkaloids is an extensive group of secondary metabolites, containing more than 12,000 different compounds [155]. Alkaloids are generally extracted from plants of the Ranunculaceae, Papaveraceae, Apocynaceae, Rutaceae, Fangke, Solanaceae, Leguminosae, and Polygonaceae families. Of course, they are also found in some animals [156]. Alkaloids’ chemical backbones have the potential to engage in interactions with an extensive array of proteins pertaining to glucose homeostasis. This has made them a highly visible and reliable candidate in the field of diabetes drug discovery, which is receiving increasing attention [157]. Some alkaloids can intervene in the insulin signal transduction pathway, reverse molecular defects resulting in insulin resistance and glucose intolerance [158]. Along with the in-depth research and application of alkaloids in the field of diabetes, the value of alkaloids in the treatment of DCM has become increasingly apparent. A total of 7 alkaloids were found to effective therapeutic intervention effects in the context of DCM. Table 3 (Ref. [159, 160, 161, 162, 163, 164, 165, 166, 167, 168, 169, 170, 171, 172, 173]) provides the basic information and mechanisms of the 7 alkaloids from recent studies on DCM, while Fig. 4 shows the chemical structures of 7 alkaloids.

**Table 3.** **Basic information and mechanisms of seven alkaloids from recent studies on DCM**.

Numbers	Terpenoid Subclass	Compounds	Molecular formula	Weight (g/mol)	Resources	Animal/Cell model	Dosage (mg/kg/d; µm)	Dosing cycle	Target/Pathways/Mechanism	Effects	Reference
1	Isoquinoline alkaloid	Berberine	$C_{20}$ $H_{18}$ ${\mathrm{NO}}_{4+}$	336.4	*Coptis chinensis* Franch.	HG-induced H9c2 cells	40	4 weeks	Activation of AMPK signaling pathway	Improved insulin resistance	[159]
						HFD + STZ-induced diabetic rats; HG-induced adult rat neonatal cardiac fibroblasts	200; 12.5, 25, 50, 100	4 weeks; 24 h	Down-regulating myocardial IGF-1 receptor-regulated MMP-2/MMP-9 expression	Decreased myocardial fibrosis, alleviated cardiac diastolic and systolic dysfunction	[160]
						STZ-induced diabetic rats	50, 100, 150	12 weeks	Down-regulation of the expression of TGF-β1 and CTGF	Decreased myocardial fibrosis	[161]
						HFD + STZ-induced diabetic rats; palmitate-induced H9c2 cells	100; 10	16 weeks; 48 h	Activation of 5′-adenosine monophosphate-activated protein kinase	Decreased myocardial fibrosis and hypertrophy	[162]
						HS, HFD + STZ-induced diabetic rats	30, 200	4, 10, 16, 22 weeks	Regulated PGC-1α and Nrf-1	Decreased myocardial fibrosis, alleviated cardiac diastolic and systolic dysfunction, regulated lipid metabolism disorders	[163]
2	Pyridine alkaloid	Matrine	$C_{15}$$H_{24}$$N_{2}$O	248.36	*Sophora flavescens* Aiton	STZ-induced diabetic rats; HG-induced mouse myocardial cells	5	10 weeks	Down-regulation of the TGF‐β/PERK signaling pathway	Anti-inflammatory, ameliorated apoptotic	[164]
						STZ-induced diabetic rats; Primary cardiac fibroblasts from LV in rats	200; 0.25, 0.5, 1.0, 1.5, 2.0, 2.5 mmol/L	10 days (drug administered before molding); 48 h	suppression activation of ATF6/calreticulin/NFAT signaling pathway	Decreased myocardial fibrosis, inhibited ECM synthesis	[165]
						STZ-induced diabetic rats	200	10 days (drug administered before molding)	Suppression TLR-4/MyD-88/caspase-8/caspase-3 signaling pathway, suppression ROS/TLR-4 signaling pathway	Anti-oxidative stress, decreased myocardial apoptosis	[166]
						STZ-induced diabetic rats	300	10 days (drug administered before molding)	Inhibition of TGF-β/Smad signaling pathways	Decreased myocardial fibrosis	[167]
						AGEs-induced diabetic rats	50, 100, 200; 0, 0.5, 1.0, 2.0 mmol/L	20 days; 24 h	Reduced ryanodine receptor 2 activity	Decreased cardiac apoptosis, attenuated calcium overload	[168]
3	Organic amine alkaloids	Betanin	$C_{24}$ $H_{26}$ $N_{2}$ $O_{13}$	550.5	*Beta vulgaris* L.	High fructose-induced diabetic rats	25, 100	60 days	Inhibition of TGF-β1 and CTGF signaling pathways, suppression NF-κB signaling pathway	Decreased myocardial fibrosis	[169]
4	Quinolizidine alkaloid	Sophocarpine	$C_{15}$$H_{22}$$N_{2}$O	246.35	*Sophora flavescens* Aiton, *Styphnolobium japonicum* (L.) Schott, etc.	STZ-induced diabetic mice; HG-induced H9c2 cells	20; 0.01–10 mM	16 weeks; 48, 96 h	Suppression NF-κB signaling pathway	Anti-inflammatory, decreased cardiac apoptosis	[170]
5	Organic amine alkaloids	Capsaicin	$C_{18}$ $H_{27}$ ${\mathrm{NO}}_{3}$	305.4	*Capsicum annuum* L.	STZ-induced diabetic rats; HG-induced mouse vascular endothelial cells	5; 1	8 weeks; 24 h	Up-regulated TRPV1/eNOS signaling pathway	Anti-oxidative stress, decreased myocardial fibrosis, ameliorated apoptosis	[171]
6	Pyridine alkaloid	Piperine	$C_{17}$ $H_{19}$ ${\mathrm{NO}}_{3}$	285.34	*Piper nigrum* L.	STZ-induced diabetic rats; HG-induced H9c2 cells	10, 20, 40	4 weeks	Regulated Bcl2, Bax/Bcl2, and caspase-3 signaling pathway	Anti-oxidative stress, decreased myocardial apoptosis	[172]
7	Isoquinoline alkaloid	Sinomenine	$C_{19}$ $H_{23}$ ${\mathrm{NO}}_{4}$	329.4	*Sinomenium acutum* (Thunb.) Rehd et Wils. (S. acutum), *Sinomenium acutum* (Thunb.) Rehd. et Wils. var. cinereum Rehd. et Wils, etc.	STZ-induced diabetic rats	30, 60, 120	10 weeks	Deactivation of NF-κB signaling pathway	Anti-inflammatory	[173]

AGE, advanced glycation end-product; ATF6, activating transcription factor 6; AMPK, AMP activated protein kinase; Bax, BCL2-Associated X; Bcl2, B-cell lymphoma-2; CTGF, connective tissue growth factor; eNOS, endothelial nitric oxide synthase; HFD, high-fat diet; HG, hyperglycemia; LV, left ventricles; MMP-2, matrix metalloproteinase-2; MMP-9 , matrix metalloproteinase-9; MyD88, myeloid differentiation factor 88; NFAT, nuclear factor of activated T cells; NF-κB, nuclear factor kappa-B; Nrf-1, nuclear respiratory factor-1; PGC1α, peroxisome proliferator-activated receptor-γ coactivator1α; PERK, protein kinase RNA-like endoplasmic reticulum kinase; ROS, reactive oxygen species; Smad, drosophila mothers against decapentaplegic protein; STZ, streptozotocin; TGF-β1, transforming growth factor-β1; TLR4, toll-like receptor 4; TRPV1, transient receptor potential vanilloid 1; DCM, diabetic cardiomyopathy; HS, high-sugar; IGF-1, insulin-like growth factor-1; ECM, extracellular matrix.

### 5.1 Berberine

*Coptis chinensis* Franch. is a THM that has been frequently used in many TCM formulas for the treatment of DM for thousands of years [174]. Berberine, the main active component of *Coptis chinensis* Franch., has been shown to have potential for the treatment of DM and its complications.

Insulin resistance is one of the most important risk factors for DCM. Berberine can improve blood sugar status via increasing insulin sensitivity in peripheral tissues. Berberine improves insulin resistance in cardiomyocytes, by increased AMPK signaling pathway activity [159]. The MMPs family is a group of enzymes involved in ECM degradation. Berberine down-regulated insulin-like growth factor-1 (IGF-1) receptor expression and MMP-2/MMP-9 levels in cardiac fibroblasts, suggesting a novel mechanism for anti-fibrotic cardioprotection of berberine in DCM [160]. In additon, berberine improves myocardial fibrosis through suppressing of TGF-β1 and connective tissue growth factor (CTGF), as well as reducing the synthesis and deposition of collagen-1 and collagen-3 [161]. Phosphatidylcholines (PCs), phosphatidylethanolamines (PEs), and sphingolipids (SMs) are closely related to the mechanism of cardiac injury during the development of DCM. The therapeutic effects of berberine on DCM is related to the interference of the metabolism of PCs, PEs, and SMs [162]. AMPK signaling pathways have attracted widespread interest as a potential therapeutic target for metabolic diseases. Berberine treatment improved cardiac dysfunction and attenuated hypertrophy in DCM. The mechanism underlying these beneficial effects is the increased activation of AMPK signaling pathways and AKT signaling pathways, along with reduced GSK3β signaling pathway activation [163].

### 5.2 Matrine

Matrine is a bioactive component of THM, such as *Sophora flavescens* and Radix *Sophorae tonkinensis*. Emerging evidence has suggested that matrine possesses anti-inflammatory, anti-oxidant stress, anti-fibrotic, anti-allergic, anti-nociceptive, hepatoprotective, cardioprotective, and neuroprotective properties [175].

The protein kinase RNA-like endoplasmic reticulum kinase (PERK) signaling pathway plays a role in ERS-mediated apoptosis. Matrine could serve as a potential anti-inflammatory and anti-apoptosis agent in the pathological processes of DCM through down-regulation of the TGF-β/PERK signaling pathway [164]. Activating transcription factor 6 (ATF6) signaling-induced myocardial fibrosis is one of the mechanisms involved in DCM. Matrine attenuated cardiac compliance, improved LV functions and inhibited myocardial fibrosis, through affecting the ATF6 signaling pathway [165]. The TLR-4/MyD88 signaling pathway is activated by excessive ROS production, which leads to cardiomyocyte apoptosis in DCM. Matrine exerts its anti-apoptotic effects by modulating TLR-4/MyD88 signaling pathway activation [166]. TGFβ1/Smad signaling also plays a role in the fibrotic process of DCM. Matrine effectively treats myocardial fibrosis by influencing the TGF-β1/Smad signaling pathway [167]. RyR2 is a ${\mathrm{Ca}}^{2+}$ release channel in the sarcoplasmic reticulum that plays a central role in cardiac excitation-contraction coupling [176]. Matrine attenuated myocardial apoptosis by regulating RyR2 [168].

### 5.3 Others

Betanin is a water-soluble alkaloid extracted from *Beta vulgaris* L. Anti-myocardial fibrosis is its key effect in DCM treatment. Betain showed significant antifibrotic effects on myocardium, which is related to inhibition of NF-κB, TGF-1, and CTGF protein expression [169].

Sophocarpine is a natural quinolizidine alkaloid derived from *Sophora flavescens* Aiton, *Styphnolobium japonicum* (L.) Schott, and other plants. Sophocarpine may be effective against DCM as it can suppress inflammation and inhibit the NF-κB signaling pathway [170].

Capsaicin is a natural protoalkaloid, derived from *Capsicum annuum* L. Capsaicin might protect against HG-induced endothelial dysfunction and DCM through the transient receptor potential vanilloid 1 (TRPV1)/eNOS signaling pathway in DCM [171].

Piperine is the source of the distinctive sharp flavor of *Piper nigrum* L. The therapeutic effects of piperine on DCM are mediated by regulation of the caspase-3, Bcl2, and Bax/Bcl2 signaling pathways. Piperine attenuates STZ-induced DCM by reducing oxidative stress, maintaining the activity of mitochondria, and preventing apoptosis [172].

Sinomenine is one of the most widely known alkaloids, due to its prominent anti-inflammatory activities. Sinomenine significantly improved cardiac function, which attributed to the de-activation of NF-κB signaling pathways and the blockade of inflammatory cytokine-mediated immune responses [173].

## 6. Quinones

**Fig. 5.**
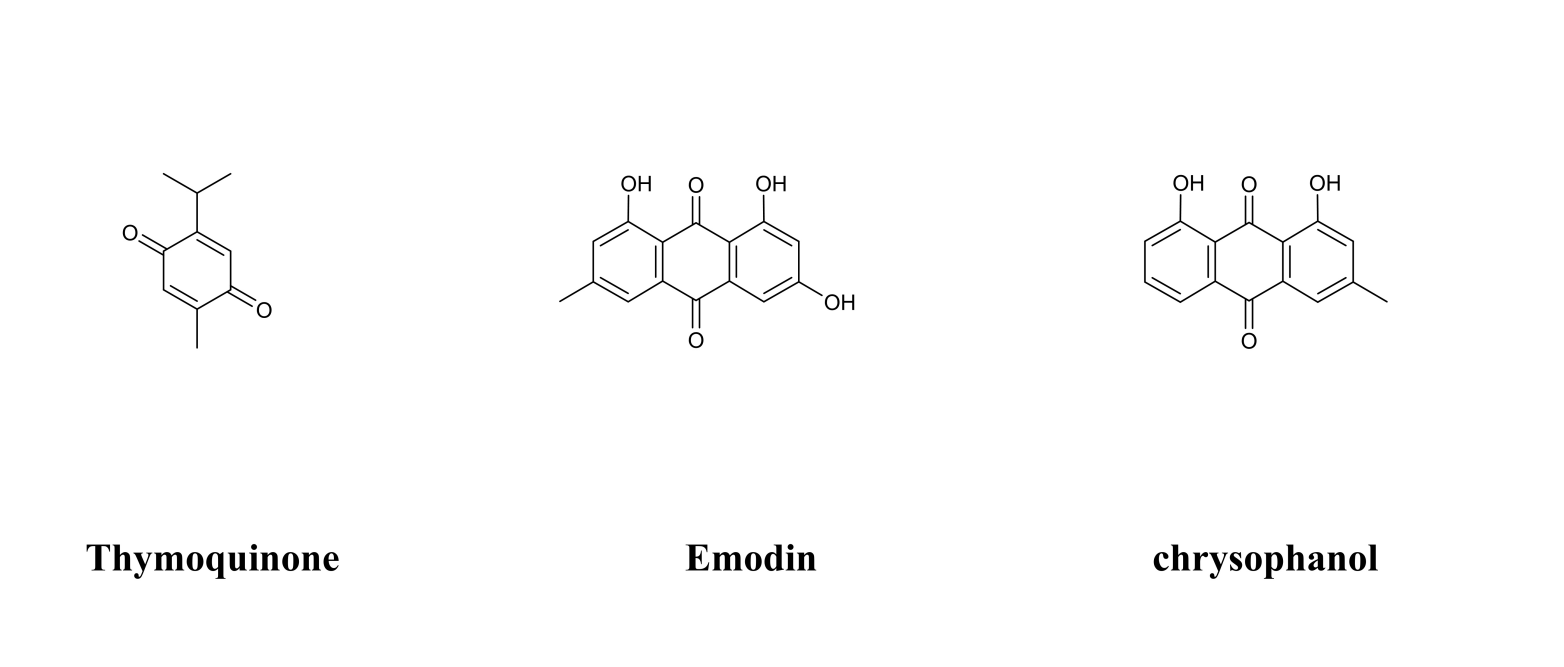
**Chemical structures of considered quinones**.

Quinones are a class of compounds widely distributed in nature, being found in a wide variety of plants as well as fungi, bacteria, and animals. Quinones are molecules comprised of a basic benzoquinone chromophore, which is an unsaturated cyclic structure with two carbonyl groups [177]. Natural quinones can be mainly divided into benzoquinones, naphthoquinones, anthraquinones, and phenanthraquinones [178]. Quinones have been reported to exhibit numerous biological activities, such as cardioprotective, antidiabetic, hepatoprotective, neuroprotective, anti-cancer, anti-inflammatory, trypanocidal, anti-viral, anti-tubercular, anti-fungal, anti-bacterial anti-filarial, anti-malarial, and so on [179]. A total of 3 quinones have been reported as having effective therapeutic intervention effects in DCM. Table 4 (Ref. [180, 181, 182, 183, 184]) provides the basic information and mechanisms of the three quinones from recent studies on DCM, while Fig. 5 shows the chemical structures of the three quinones.

**Table 4.** **Introduction to the basic information and mechanisms of 3 quinones on DCM from recent studies**.

Number	Terpenoid Subclass	Compounds	Molecular formula	Weight (g/mol)	Resources	Animal/Cell model	Dosage (mg/kg/d; µm)	Dosing cycle	Target/Pathways/Mechanism	Effects	Reference
1	Benzoquinone	Thymoquinone	$C_{10}$ $H_{12}$ $O_{2}$	164.2	*Nigella damascena* L.	STZ-induced diabetic rats; HG-induced H9c2 cells	50	12 weeks	Up-regulation of Nrf-2 signaling pathways	Anti-oxidative stress, anti-inflammatory	[180]
						STZ-induced diabetic rats	50	30 days	Up-regulation of PI3K/AKT signaling pathways	Anti-oxidative stress, anti-inflammatory, decreased myocardial apoptosis	[181]
						STZ-induced diabetic rats	20	5 weeks	/	Anti-oxidative stress, anti-inflammatory	[182]
2	Anthraquinone	Emodin	$C_{15}$ $H_{10}$ $O_{5}$	270.24	*Rheum palmatum* L., *Reynoutria japonica* Houtt., and *Pleuropterus multiflorus* (Thunb.) Nakai.	High fructose + HFD + STZ-induced diabetic rats	50, 100	16 weeks	Up-regulation of AKT/GSK-3β signaling pathways	Regulated glycolipid metabolism	[183]
3	Anthraquinone	chrysophanol	$C_{15}$ $H_{10}$ $O_{4}$	254.24	*Rheum palmatum* L., *Senna tora* (L.) Roxb., *Aloe vera* (L.) Burm. f., etc.	HFD-induced Nrf-2-knockout (Nrf-2-/-) DCM mice, HG-induced H9c2 cells	25, 50; ① 0, 10, 20, 40, 80, 160 , 320; ② 320	20 weeks; ① 24 h, ② 0, 6,12, 24, 36, 48, and 72 h	Up-regulation of Nrf-2 signaling pathways	Decreased myocardial fibrosis, anti-oxidative stress, anti-inflammatory	[184]

①, ②: This distinction is made because the study used two dosing methods and contents for cellular intervention. Treatment of H9c2 cells with different concentrations of chrysophanol as indicated (0, 10, 20, 40, 80, 160 and 320 µm) for 24 h. In addition, the cells were cultured with chrysophanol at 320 µm for different time (0, 6, 12, 24, 36, 48 and 72 h). After various treatments, all cells were harvested for cell viability. AKT, protein kinase B; GSK-3β, glycogen synthase kinase 3β; HFD, high-fat diet; HG, hyperglycemia; Nrf-2, nuclear factor erythroid 2-related factor 2; PI3K, phosphoinositide 3-Kinase; STZ, streptozotocin; DCM, diabetic cardiomyopathy.

### 6.1 Thymoquinone

Thymoquinone, a phytochemical compound obtained from *Nigella sativa*, has received attention for its anti-inflammatory, analgesic, anti-cancer, anti-oxidant, and anti-pyretic activities [185]. Thymoquinone diminished oxidative damage by improving the anti-oxidant power of cardiac muscle, consequently protecting the cardiac muscles and alleviating the inflammatory process. The mechanism of action in this research was mainly through up-regulation of Nrf-2 signaling pathways [180]. The protective impact of thymoquinone enhances cardiovascular performance while mitigating oxidative stress, inflammation, and apoptosis through mediation of the PI3K/Akt signaling pathway [181]. Thymoquinone is a pharmacological agent that has potential for the treatment of DCM, and its healing power further increases even more when combined with β-aminoisobutyric acid [182]. This provides a novel idea for the pharmacological study of thymoquinone.

### 6.2 Others

Emodin is a natural anthraquinone derivative that occurs in many widely used herbs, such as *Rheum palmatum* L., *Reynoutria japonica* Houtt., and *Pleuropterus multiflorus* (Thunb.) Nakai. DM and its cardiovascular complications are closely related to impairment of the AKT/GSK-3β signaling pathway. Emodin can protect against DCM by regulating the AKT/GSK-3β signaling pathway [183].

Chrysophanol is a naturally occurring anthraquinone found in various herbs, including *Rheum palmatum* L., *Senna tora* (L.) Roxb., and *Aloe vera* (L.) Burm. f. The anti-oxidant stress, anti-inflammatory, and anti-fibrosis effects of chrysophanol can be explained by its regulation of Nrf-2 signaling pathway in DCM [184].

## 7. Others

**Fig. 6.**
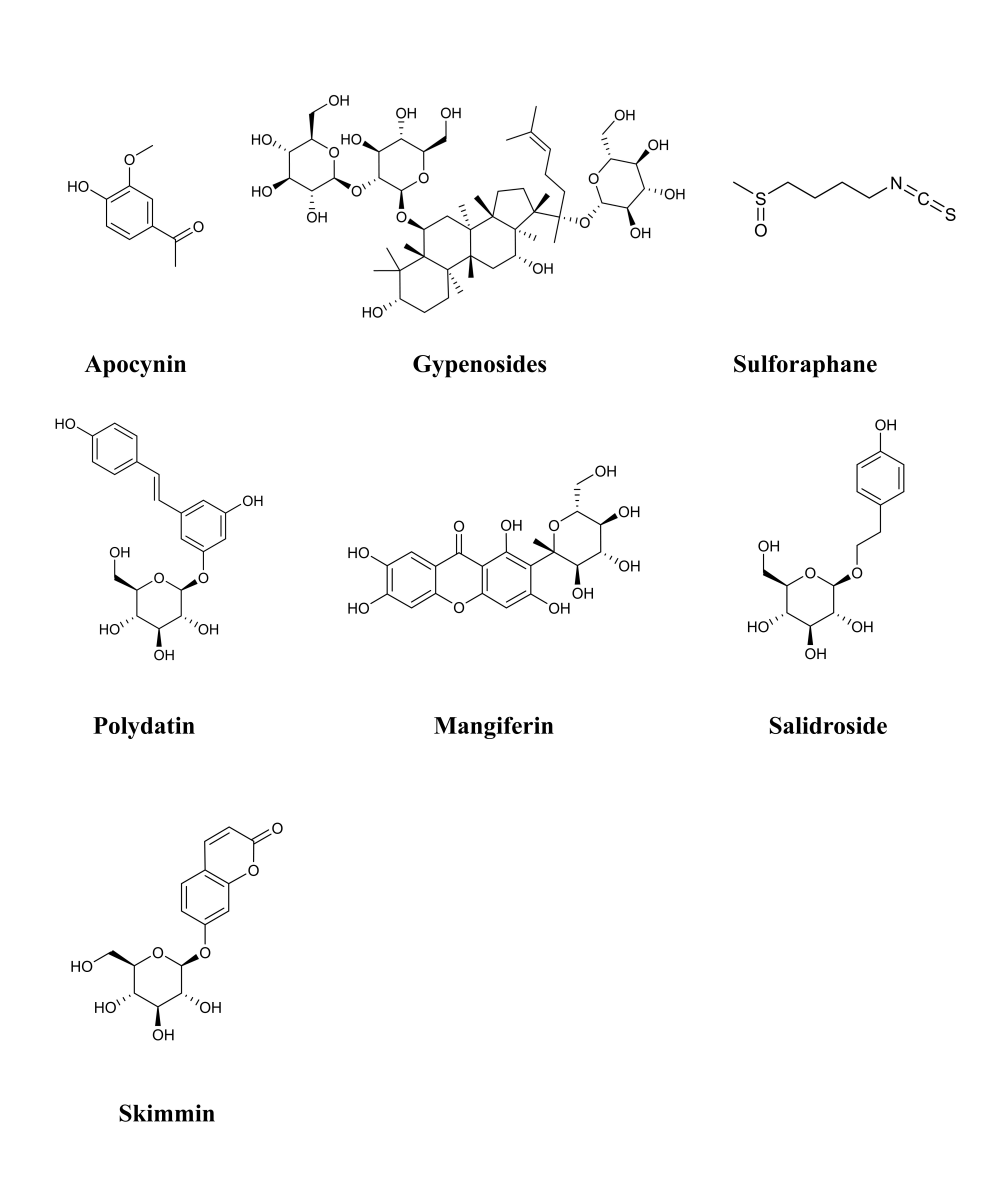
**Chemical structures of other natural compounds**.

In addition to the flavonoids, terpenoids, alkaloids, and quinones discussed above, many other kinds of natural products can also be used to treat DCM. Table 5 (Ref. [186, 187, 188, 189, 190, 191, 192]) provide the basic information and mechanisms of seven additional natural compounds derived from recent studies on DCM, while Fig. 6 shows the chemical structures of the seven natural products.

**Table 5.** **Basic information and mechanisms of seven additional natural compounds from recent studies on DCM**.

Number	Flavonoid Subclass	Compounds	Molecular formula	Weight (g/mol)	Resources	Animal/Cell model	Dosage (mg/kg/d; µm/24 h)	Dosing cycle	Target/Pathways/Mechanism	Effects	Reference
1	Glycoside	Apocynin	$C_{9}$ $H_{10}$ $O_{3}$	166.17	*Iris tectorum* Maxim., *Cannabis sativa* L., etc.	HFD + STZ-induced diabetic mice; HG-induced NRCMs and CFs	10; 400	3 months, 48 h	Suppression of ASK1-p38/JNK signaling pathways	Anti-oxidative stress, decreased myocardial apoptosis, decreased myocardial fibrosis, attenuated cardiomyocyte hypertrophy	[186]
2	Glycoside	Gypenosides	$C_{48}$ $H_{82}$ $O_{19}$	963.2	*Gynostemma pentaphyllum* (Thunb.) Makino	STZ-induced diabetic rats; HG-induced H9c2 cells	200; 100, 200, 400 mg/L	8 weeks; 48 h	Inhibition of NLRP3 inflammasome activation	Anti-inflammatory	[187]
3	Glycoside	Sulforaphane	$C_{6}$ $H_{11}$ ${\mathrm{NOS}}_{2}$	177.3	*Brassica oleracea var.* italica Plenck, *Brassica oleracea* L., etc.	HFD + STZ-induced diabetic mice	0.5	3 months	Activation of AMPK/AKT/GSK3β signaling pathway	Anti-oxidant stress, anti-inflammatory, decreased myocardial fibrosis and hypertrophy	[188]
4	Glycoside	Polydatin	$C_{20}$ $H_{22}$ $O_{8}$	390.4	*Reynoutria japonica* Houtt.	Sirt3 knockout (Sirt3-/-) mice; HG-induced primary neonatal mouse ventricular cardiomyocyte	7.5; 10	28 d; 48 h	Up-regulated SIRT3 signaling pathway	Increased autophagy, improved mitochondrial bioenergetics	[189]
5	Glycoside	Mangiferin	$C_{19}$ $H_{18}$ $O_{11}$	422.3	*Anemarrhena asphodeloides* Bunge, *Mangifera indica* L., etc.	HFD + STZ-induced diabetic rats	20	16 weeks	De-activation of NF-κB translocation	Anti-inflammatory, decreased myocardial fibrosis	[190]
6	Phenylpropanoid	Salidroside	$C_{14}$ $H_{20}$ $O_{7}$	300.3	*Rhodiola rosea* L.	Diabetic db/db mice; AGEs-induced H9c2 cells	0.025, 0.05; 0.1, 1, 10	12 weeks; 6 h	Activation of AKT/Nrf-2/HO-1 signaling pathway	Decreased myocardial fibrosis, decreased cardiac apoptosis	[191]
7	Phenylpropanoid	Skimmin	$C_{15}$ $H_{16}$ $O_{8}$	324.28	*Artemisia caruifolia* Buch.-Ham. ex Roxb., *Astragalus membranaceus var. mongholicus* (Bunge) P. K. Hsiao, etc.	STZ-induced diabetic rats; HG-induced primary neonatal cardiomyocytes from rats	15, 30; 2, 10	16 weeks; 24 h	Suppression of NLRP3 inflammasome activation	Anti-inflammatory, anti-oxidative stress, increased autophagy, inhibited pyroptosis	[192]

AKT, protein kinase B; ASK1, apoptosis signal regulating kinase 1; CFs, cardiac fibroblasts; GSK-3β, glycogen synthase kinase 3β; HFD, high-fat diet; HG, hyperglycemia; HO-1, heme oxygenase-1; JNK, c-Jun N-terminal protein kinase; NF-κB, nuclear factor kappa-B; Nrf-2, nuclear factor erythroid 2-related factor 2; NLRP-3, nucleotide-binding oligomerization domain-like receptor with a pyrin domain 3; NRCMs, Neonatal rat cardiomyocytes; SIRT3, silent information regulator 3; DCM, diabetic cardiomyopathy; STZ, streptozotocin; AMPK, AMP activated protein kinase.

Apocynin is a naturally occurring glycoside found in *Iris tectorum* Maxim. and *Cannabis sativa* L. Apocynin may act as a potential inhibitor of apoptosis signal regulating kinase (ASK), and attenuated cardiomyocyte hypertrophy, myocardial fibrosis, and cardiac dysfunction in by inhibiting the ASK1-p38/JNK signaling pathways [186].

Gypenosides are the main active ingredients of *Gynostemma pentaphyllum* (Thunb.) Makino, which is a TCM commonly used in China. Gypenoside inhibited HG-induced myocardial damage through anti-inflammatory effects. The mechanism of action may be the inhibition of NLRP3 inflammasome activation and NLRP3 [187].

Sulforaphane is mainly present in the sprouts of many cruciferous vegetables, belonging to the isothiocyanate family. AMPK is indispensable for the sulforaphane-induced prevention of cardiomyopathy in T2DM, and the activation of Nrf-2 by sulforaphane is mediated by the AMPK/AKT/GSK3β signaling pathways. Through this mechanism, it presented anti-oxidant stress, anti-inflammatory, decreased myocardial fibrosis, and hypertrophy [188].

Polydatin is a glycoside isolated from *Reynoutria japonica* Houtt. Polydatin improved cardiac dysfunction, increased autophagy flux, and regulated mitochondrial bioenergetics, by up-regulating Sirt3 signaling pathways in DCM [189].

Mangiferin, a bioactive glycoside compound present in mango, has been reported to be valuable in treating DCM. Mangiferin ameliorated DCM by preventing the release of inflammatory cytokines, inhibiting ROS accumulation, reducing AGE/RAGE production, and regulating NF-κB nuclear translocation [190].

Salidroside is isolated from *Rhodiola rosea* L., which has been used for a long time as an adaptogen in TCM. Salidroside treatment protects against cardiomyocyte apoptosis and ventricular remodeling in the hearts of diabetic patients. This cardio-protective effect of salidroside is dependent on activation of the AKT/Nrf-2/HO-1 signaling pathways [191].

Skimmin, a natural coumarin derivative, has been shown to possess protective effects against experimental DCM. Skimmin has the potential to prevent DCM by reducing NLRP3 inflammasome activation and promoting autophagy in heart tissues, as well as potentially inhibiting pyroptosis [192].

## 8. Conclusions

A total of 72 natural compounds were discussed in this study, divided into five categories based on their chemical structure characteristics: Flavonoids, terpenoids, alkaloids, quinones, and others. Flavonoids are the largest group of natural products reported for the treatment of DCM, with a total of 36 flavonoids retrieved. The number of terpenoids in this study ranked second, with a total of 19 species. The quantitative distribution of various types of natural products also provides scope and guidance for the future exploration and development of new drugs for use in the treatment of DCM.

Various studies have found that the efficacy of a natural product in the treatment of DCM mainly depends on its properties in terms of anti-oxidant stress, anti-inflammatory, regulation of programmed cell death (including apoptosis, necroptosis, pyroptosis, and autophagy), regulation of glucose and lipid metabolism, regulation of ${\mathrm{Ca}}^{2+}$ homeostasis, anti-fibrosis, and protection of mitochondria and ER function and structure. Oxidative stress is regarded as a significant factor in the pathogenesis of DCM. A total of 50 types of natural products were reported as having anti-oxidant effects in this study. Targeting inflammatory cascades to prevent DCM may have potential benefits due to the impact of inflammation on the onset and development of DCM [193]. A total of 47 types of natural products were identified as having anti-inflammatory effects. The prevention and treatment of myocardial fibrosis are important in preventing the occurrence of further HF. A total of 42 types of natural products were identified as having anti-fibrotic effects.

Natural products have the advantages of being multi-pathway, multi-link, and multi-target agents in the treatment of DCM, and can play various roles through different signaling pathways. However, there is some commonality in the intervention signaling pathways when treating the same disease. NF-κB has been long proposed as a potential target for the therapy of inflammatory diseases. There were 22 types of natural products exhibiting anti-inflammatory effects through the NF-κB signaling pathway. Nrf-2 is a truly pleiotropic transcription factor that regulates many cellular processes. Through the Nrf-2 signaling pathway, 19 types of natural products considered here presented the myocardial protective effects of anti-oxidative stress, anti-inflammation, anti-apoptosis, and myocardial fibrosis inhibition. TGF-β plays an important role in the pathogenesis of cardiac remodeling and myocardial fibrosis. A total of 12 types of natural products inhibited the progression of myocardial fibrosis in DCM through the TGF-β signaling pathway.

Overall, this paper provided a brief overview of the main directions of natural product research related to DCM to date, as well as pointing out promising avenues for future research. It must be noted that studies existing in the literature have been restricted to animal and cell experiments, with a lack of clinical research. Further clinical research utilizing natural products could provide more insight into the effectiveness of their complicated pharmacological properties, enabling natural products to be used safely and efficiently.

## Abbreviations

ADSC, adipose-derived stem cells; AGEs, advanced glycation end-products; AKT, protein kinase B; AMPK, AMP activated protein kinase; ARE, antioxidant response element; ASK1, apoptosis signal regulating kinase 1; ATF6, activating transcription factor 6; ATP, adenosine triphosphate; Bax, BCL2-Associated X; Bcl2, B-cell lymphoma-2; BNP, brain natriuretic peptide; CFs, cardiac fibroblasts; cGMP, cyclic guanosine monophosphate; CHD, coronary heart disease; CK-MB, serum creatine kinase MB isozyme; CRP, reactive protein; CTGF, connective tissue growth factor; CVD, cardiovascular disease; CXCR4, C-X-C chemokine receptor type 4; Cyt c, cytochrome c; DCM, diabetic cardiomyopathy; DKD, diabetic kidney disease; DM, diabetes mellitus; EC, (-)-epicatechin; ECG, (-)-epicatechin-3-gallate; ECM, extracellular matrix; ECs, endothelial cells; ED, endothelial dysfunction; EETs, epoxyeicosatrienoic acids; EGC, (-)-epigallocatechin; EGCG, (-)-epigallocatechin-3-gallate; eNOS, endothelial nitric oxide synthase; ER, endoplasmic reticulum; ERK, extracellular signal-regulated kinase; ERK1/2 , extracellular signal-regulated kinase1/2; ERS, endoplasmic reticulum stress; ESC, European Society of Cardiology; FA, fatty acid; FFA, free fatty acids; GP, glycogen phosphorylase; GS, glycogen synthase; GSK-3β, glycogen synthase kinase 3β; HC, high cholesterol diet; HDAC4, histone deacetylase 4; HF, heart failure; HFD, high-fat diet; HG, hyperglycemia; HO-1, heme oxygenase-1; HS, high-sugar; IDDM, insulin-dependent diabetes mellitus; IDH2, isocitrate dehydrogenase 2; IGF-1, insulin-like growth factor-1; IkB-α, NF-κB inhibitor alpha; IL-1β, interleukin 1β; IRS, insulin receptor substrate; JNK, c-Jun N-terminal protein kinase; KATP, ATP-sensitive K(+); Keap1, kelch-like ECH-associated protein 1; LC-3, light chain 3; LDH, lactate dehydrogenase; LV, left ventricles; LVH, left ventricular hypertrophy; MAPK, mitogen-activated protein kinases; MEF2, myocyte enhancer factor 2; miRNA, MicroRNA; MMP-2, matrix metalloproteinase-2; MMP-9 , matrix metalloproteinase-9; MMPs, matrix metalloproteinases; mPT, mitochondria permeability transition; mTOR, mammalian target of rapamycin; MyD88, myeloid differentiation factor 88; NAD+, nicotinamide adenine; NCX, ${\mathrm{Na}}^{+}$/${\mathrm{Ca}}^{2+}$ exchanger; Neat1, long non-coding RNA nuclear paraspeckle assembly transcript 1; NFAT, nuclear factor of activated T cells; NF-κB, nuclear factor kappa-B; Nix, NIP3-like protein X; NLRP3, nucleotide-binding oligomerization domain-like receptor with a pyrin domain 3; NOS, nitric oxide synthase; NOX, NADPH oxidase; NOX4, NADPH oxidase 4; NRCMs, Neonatal rat cardiomyocytes; Nrf-1, nuclear respiratory factor-1; Nrf-2, nuclear factor erythroid 2-related factor 2; NRVMs, neonatal rat ventricular myocytes; O-GlcNAcylation, O-linked-N-acetylglucosaminylation; pAKT, phosphorylated-Serine473-AKT; PCD, programmed cell death; PCs, phosphatidylcholines; PDE5, phosphodiesterase 5; PERK, protein kinase RNA-like endoplasmic reticulum kinase; PEs, phosphatidylethanolamines; PGC-1α, peroxisome proliferator-activated receptor-gamma coactivator-1α; PI3K, phosphoinositide 3-Kinase; PKB, protein kinase B; PKG, protein kinase G; PKR, protein kinase R; PPARs, peroxisome proliferator-activated receptors; PPARγ, peroxisome proliferator-activated receptor γ; PPARδ, peroxisome proliferator-activated receptor δ; RAGE, advanced glycosylation end-product receptor; ROS, reactive oxygen species; RyR2, ryanodine receptor type 2; SDF1, stromal cell-derived factor-1; SERCA2a, sarco/endoplasmic reticulum calcium ATPase; sGC, soluble guanylate cyclase; SIRT1, silent information regulator 1; SIRT3, silent information regulator 3; SIRT5, silent information regulator 5; Smad, drosophila mothers against decapentaplegic protein; SMs, sphingolipids; STAT3, signal transducer and activator of transcription 3; STZ, streptozotocin; T1DM, type 1 diabetes mellitus; T2DM, type 2 diabetes mellitus; TAC, transverse aortic constriction; TCM, traditional Chinese medicine; TGF-β, transforming growth factor-β; TGF-β1, transforming growth factor-β1; THM, Traditional herbal medicine; TLR4, toll-like receptor 4; TNF-α, tumor necrosis factor α; TRPV1, transient receptor potential vanilloid 1; tTG, tissue transglutaminase; ULK1, Unc-51-like autophagy activating kinase 1; VCAM-1, vascular cell adhesion molecule 1; α-SMA, α-smooth muscle actin.
